# Purinergic System Transcript Changes in the Dorsolateral Prefrontal Cortex in Suicide and Major Depressive Disorder

**DOI:** 10.3390/ijms26051826

**Published:** 2025-02-20

**Authors:** Smita Sahay, Anna E. Lundh, Roshan P. Sirole, Robert E. McCullumsmith, Sinead M. O’Donovan

**Affiliations:** 1 Department of Neurosciences and Psychiatry, University of Toledo College of Medicine and Life Sciences, Toledo, OH 43614, USA; smita.sahay@rockets.utoledo.edu (S.S.);; 2 Neurosciences Institute, ProMedica, Toledo, OH 43606, USA; 3 Department of Biological Sciences, University of Limerick, Castletroy, V94 T9PX Limerick, Ireland

**Keywords:** purinergic system, suicide, major depressive disorder, dorsolateral prefrontal cortex, cortical layers, postmortem, transcript

## Abstract

Suicide is a major public health priority, and its molecular mechanisms appear to be related to imbalanced purine metabolism in the brain. This exploratory study investigates purinergic gene expression in the postmortem dorsolateral prefrontal cortex (DLPFC) tissue isolated from subjects with major depressive disorder (MDD) who died by suicide (MDD-S, *n* = 10), MDD subjects who did not die by suicide (MDD-NS, *n* = 6) and non-psychiatrically ill controls (CTL, *n* = 9–10). Purinergic system transcripts were assayed by quantitative polymerase chain reactions (qPCR) in superficial and deep gray matter as well as white matter DLPFC cortical layers using laser microdissection (LMD). Across all subjects, regardless of sex, *P2RY12* (F_(2,23)_ = 5.40, *p* = 0.004) and *P2RY13* (KW statistic = 11.82, *p* = 0.001) transcript levels were significantly greater in MDD-S compared to MDD-NS subjects. Several other perturbations were observed in the white matter tissue isolated from females: *NT5E* (F_(2,10)_ = 13.37, *p* = 0.001) and *P2RY13* (F_(2,9)_ = 3.99, *p* = 0.011, controlled for age) transcript expression was significantly greater in MDD-S vs. MDD-NS female groups. *ENTPD2* (F_(2,10)_ = 5.20, *p* = 0.03), *ENTPD3* (F_(2,10)_ = 28.99, *p* < 0.0001), and *NT5E* (F_(2,10)_ = 13.37, *p* = 0.001) were among the transcripts whose expression was significantly elevated in MDD-S vs. CTL female groups. Transcripts that exhibited significantly altered expression in the superficial and deep gray matter included *ENTPD2*, *NT5E*, *PANX1*, and *P2RY13* (*p* ≤ 0.05). Our medication analysis revealed that the expression of these transcripts was not significantly altered by antidepressants. This is the first study to holistically quantify the purinergic metabolic pathway transcripts in suicide and MDD utilizing human postmortem brain tissue. Our preliminary findings support evidence implicating changes in purinergic P2 receptors in the brain in suicide and provide support for broader purinergic system dysregulation in mood disorders.

## 1. Introduction

Suicide, the intentional self-infliction of death, is the 11th-ranking cause of mortality in the United States affecting individuals of all ages and remains a significant public health concern [1,2]. Pharmacologic treatments often target psychiatric illnesses that are risk factors for suicide. For example, esketamine has been approved by the United States Food and Drug Administration (FDA) for individuals with major depressive disorder (MDD) who have acute, active suicidal ideation with intent [3]. For patients with chronic suicidality, clozapine remains the only FDA-approved anti-suicidal medication, with its use primarily for people with schizophrenia and schizoaffective disorder [4]. Medications designed to treat suicide risk in the absence of an underlying psychiatric illness do not exist. Suicidality has been linked with agitation, aggression, and poor impulse control independent of a psychiatric illness [5] and has therefore been suggested as a distinct diagnosis within the classification of mental disorders [6,7], but this remains a topic of debate. Nonetheless, the etiology of MDD and suicide are unknown, although they are speculated to be a combination of genetic, environmental, and stress factors [8]. Growing evidence suggests that the purinergic system is implicated in the neurobiology of suicide in the context of psychiatric disorders like MDD [9], as well as in behavioral phenotypes such as impulsivity and aggressiveness [10], but less is known about this system in the human brain. Due to the practical and ethical constraints in researching suicide, the study of postmortem brain tissue from individuals who have died by suicide provides a unique and valuable opportunity to explore the underlying pathophysiological changes in the brain.

The purinergic system as a whole involves the release and effects of the energy-carrying molecule adenosine triphosphate (ATP) and its breakdown products adenosine diphosphate (ADP), adenosine monophosphate (AMP), and the purine ribonucleoside, adenosine [11]. These molecules are part of a larger, complex network composed of enzymes, channels, transporters, and receptors that regulate neurotransmission, synaptic plasticity, and cell signaling [12]. Adenosine and non-adenosine nucleosides such as guanosine, inosine, and uridine modulate physiological and pathological processes in the brain related to sleep, pain, memory, and mood [13]. Disruption of the purinergic system is associated with various psychiatric phenotypes. For instance, magnetic resonance spectroscopy (MRS) studies report a correlation between depleted ATP levels in frontal cortical brain regions and the presence of MDD in adult patients [14,15,16].

One part of the purinergic system involves the extracellular catabolism of ATP to adenosine. The ratio of ATP to adenosine is tightly regulated in the extracellular space via ectonucleotidases—ectoNTPDases (*ENTPD*), ectoNPPases (*ENPP*), alkaline phosphatases (ALPL), and ecto-5′-nucleotidases (*NT5E*)—a family of enzymes that dephosphorylate ATP to prevent prolonged agonist exposure and receptor desensitization [9,17]. Another level of regulation is via the pannexin 1 (*PANX1*) channels on neuronal and glial cell membranes, which release ATP extracellularly [18]. In addition to the extracellular formation of adenosine via ectonucleotidases, adenosine may also be released to the extracellular space (or be taken up to nerve terminals) via bidirectional equilibrative and concentrative nucleoside transporters (*ENT*s and *CNT*s) [17]. Further catabolism of adenosine to inosine, and eventually to uric acid, is mediated by adenosine deaminase (*ADA*) intra- and extra-cellularly [17].

ATP and adenosine form a separate signaling system at the level of receptors. The actions of adenosine are regulated by metabotropic purinergic P1 receptors, which are subdivided into A_1_, A_2A_, A_2B_, and A_3_ with the highest affinity receptors being the A_1_ (*ADORA1*) and A_2A_ (*ADORA2A*) subtypes [19,20]. Adenosine formed from adenosine nucleotides preferentially activates A_2A_ whereas adenosine released by nucleoside transporters (*ENT* and *CNT*) preferentially activates A_1_ [21]. In MDD, animal model studies have reported that modulating *ADA*, A_1_, and/or A_2A_ receptors induces depression-like endophenotypes such as learned helplessness and behavioral despair in rodents [22,23]. A_2A_ receptor antagonists show inhibitory effects on depressive symptoms, improve anxiety-like behaviors in mice [24], and prevent early stress-induced synaptic modifications, possibly relieving chronic stress associated with depression [25]. While MDD is a significant risk factor for suicide, suicidal ideation among individuals with MDD or suicide itself cannot be observed or modeled in animals. However, epidemiological findings in suicide support animal models of MDD, showing that consumption of caffeine, a nonselective adenosine receptor antagonist [26], is negatively correlated with completed suicide attempts [27].

The actions of di- and tri- phosphates such as ATP and ADP are regulated by purinergic P2 receptors, which are further subclassified as P2X or P2Y receptors. Ionotropic P2X receptors are ATP-gated ion channels permeable to sodium, potassium, and calcium cations whereas metabotropic P2Y receptors are directly activated by ATP and ADP [19,28]. Data on P2 purinergic receptor involvement in MDD are relatively limited, but the P2RX7 receptor is of interest due to genome-wide association studies (GWAS) linking at least 12 single nucleotide polymorphisms (SNPs) in *P2RX7* to mood disorders [29]. Postmortem and transcriptomic studies within the last three years have reported the involvement of altered P2RY12, P2RY13, and P2RY14 receptors in the frontal cortical brain regions of individuals who have died by suicide [30,31]. Given these findings, further investigations are warranted to better understand these perturbations.

The enzymes, channels, transporters, and receptors discussed thus far that constitute the purinergic system are broadly expressed throughout the central nervous system (CNS) and mediate a range of normal and pathological functions [17]. To better understand this system in the brain, we assayed the gene expression of 16 purinergic system components in postmortem human dorsolateral prefrontal cortex (DLPFC)—a region associated with behavior and emotional deficits in general [32,33] as well as aggression control and suicidality [34]—of subjects diagnosed with MDD, comparing those who died by suicide (MDD-S) to those who did not (MDD-NS), as well as to non-psychiatrically ill control subjects (CTL).

In this exploratory study, we isolated superficial (layers II–III) and deep (layers V–VI) gray matter as well as white matter DLPFC cortical layers utilizing laser microdissection (LMD) to enhance the resolution of purinergic transcript detection. Traditional whole brain homogenates average mRNA signals across mixed cell layers and types, which dilutes mRNA-level changes [35]. By focusing on cortical layers, we capture more precise mRNA profiles, reflecting the distinct physiological roles of each layer: cortical communication and subcortical projections in the superficial and deep layers, and corticocortical communication in the white matter [36,37]. LMD thus enables more accurate information about layer-specific cortical functions and cell type-specific processes [38]. We assayed 16 purinergic transcripts in total, including components of the extracellular ATP catabolic portion of the purinergic system (*n* = 9) and purinergic P1 and P2 receptors (*n* = 7).

The purinergic system’s critical role in maintaining CNS function and modulating mood- and impulsivity-related behaviors emphasizes the importance of understanding transcript dysfunction in this pathway. While preliminary, this study provides foundational insights into purinergic system dysregulation and its role in suicide and MDD. These findings aim to guide extensive future studies of purinergic signaling aberrations in neuropsychiatric illnesses.

## 2. Results

Transcript expression levels of extracellular ATP catabolic purinergic transcripts (*n* = 9) and purinergic receptors (*n* = 7) were measured in postmortem DLPFC tissue from superficial gray matter, deep gray matter, and white matter cell layers, with each layer analyzed independently. Expression levels were compared among CTL, MDD-NS, and MDD-S groups to identify diagnostic category-associated differences. Analyses were conducted for all subjects (females and males combined), as well as for females and males separately, to assess sex-dependent changes. The impact of antidepressant medication on transcript expression was also evaluated to determine treatment-related effects. Transcripts with no significant expression changes in any comparison of interest in at least one cell layer are not shown. Significant findings (*p* ≤ 0.05) are detailed in the following subsections. The *p*-values reported below have been adjusted using post hoc analysis to account for multiple comparisons. All data and statistical results are summarized in Supplementary Tables: Table S5 (all subjects: one-way and two-way ANOVA results), Table S6 (females and males separately, one-way ANOVA results), and Table S7 (medication analysis, *t*-test results).

### 2.1. Purinergic Transcript Expression Changes Exclusive to Suicide Pathology

Here, we report significant differences found between the MDD-S and MDD-NS groups to highlight purinergic changes that are specific to the pathophysiology of suicide. Significant differences between other disease groups are reported in the following subsections.

**Figure 1:**
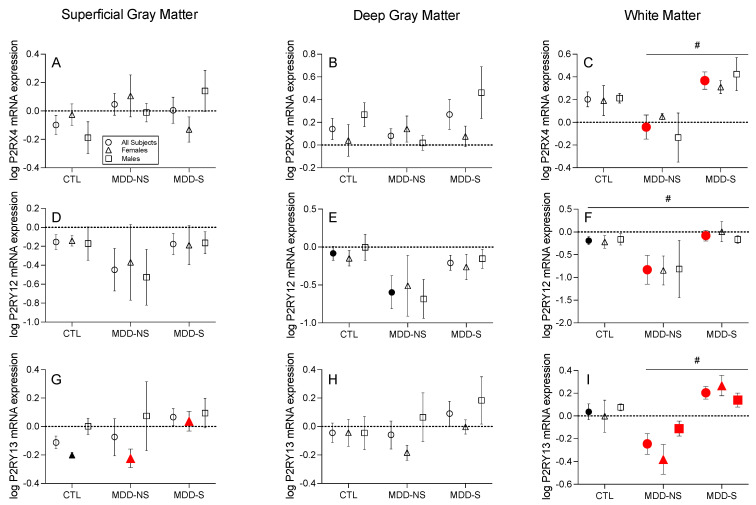
Transcript Expression Changes among Purinergic Receptors. *P2RX4* mRNA expression was significantly greater in MDD-S vs. MDD-NS all subject groups in the (**C**) white matter. (**F**) *P2RY12* mRNA expression was significantly greater in the MDD-S vs. MDD-NS all subjects group in the white matter. (**G**) *P2RY13* mRNA expression was significantly greater in the MDD-S vs. MDD-NS females in the superficial gray matter. (**I**) *P2RY13* mRNA expression was significantly greater in the MDD-S vs. MDD-NS subjects in all three groups in the white matter: all subjects, females only, and males only. Transcripts shown include those with at least one suicide-specific significant difference (i.e., between MDD-S and MDD-NS) in at least one comparison group (all subjects, females only, or males only) in at least one cell layer (superficial, deep, or white matter). If a suicide-specific significant difference was identified, data for that transcript are displayed across all cell layers. Of the displayed transcripts, (**E**,**F**) *P2RY12* and (**G**,**I**) *P2RY13* exhibited additional significant differences between either MDD-NS or MDD-S compared to CTL subjects. These findings are discussed later in the context of Figure 3 but shown here for completeness. No significant differences between groups observed in (**A**,**B**,**D**,**H**). Points show the mean ± standard error of the mean (SEM) for all subjects (circles, *n* = 6–10), females only (triangles, *n* = 3–5), and males only (squares, *n* = 3–5). Filled symbols indicate significantly different transcript expression as determined by analysis of variance (ANOVA) and post hoc tests. Red shapes indicate suicide-specific significant differences and black shapes indicate non-suicide-specific significant differences. # indicates a significant main effect of disease in the all subjects comparison, determined by two-way ANOVA. Data and results of statistical tests are reported in Supplementary Tables S5 and S6. Abbreviations: CTL, control; MDD-NS, major depressive disorder—non-suicide; MDD-S, major depressive disorder—suicide; *P2RX4*, Purinergic receptor P2X 4; *P2RY12*, purinergic receptor P2Y 12; *P2RY13*, purinergic receptor P2Y 13.

*Purinergic Receptor Transcripts:* Among the purinergic P2X receptors, *P2RX4* mRNA expression was significantly greater in the MDD-S compared to MDD-NS all subject analysis in the white matter (Kruskal–Wallis (KW) nonparametric test: KW statistic = 8.81, *p* = 0.01), with a significant main effect of disease observed (two-way ANOVA: F_(2,14)_ = 4.46, *p* = 0.03, Figure 1C). Among the purinergic P2Y receptors, *P2RY12* mRNA expression was significantly greater in the MDD-S compared to the MDD-NS all subjects group in the white matter, with a significant main effect of disease observed (two-way ANOVA: F_(2,14)_ = 5.82, *p* = 0.01, Figure 1F). *P2RY13* mRNA expression was significantly greater in the MDD-S compared to MDD-NS females in the superficial gray matter (one-way ANOVA: F_(2,10)_ = 7.11, *p* = 0.03, Figure 1G). *P2RY13* mRNA expression was significantly greater in the MDD-S compared to MDD-NS subjects in all three comparison groups in the white matter: all subjects (ANCOVA: F_(2,22)_ = 4.62, *p* = 0.001, after controlling for the effect of age), with a significant main effect of disease observed (two-way ANOVA: F_(2,14)_ = 6.54, *p* = 0.01), females only (ANCOVA: F_(2,9)_ = 3.99, *p* = 0.01, after controlling for the effect of age), and males only (one-way ANOVA: F_(2,10)_ = 4.99, *p* = 0.03) (Figure 1I).

**Figure 2:**
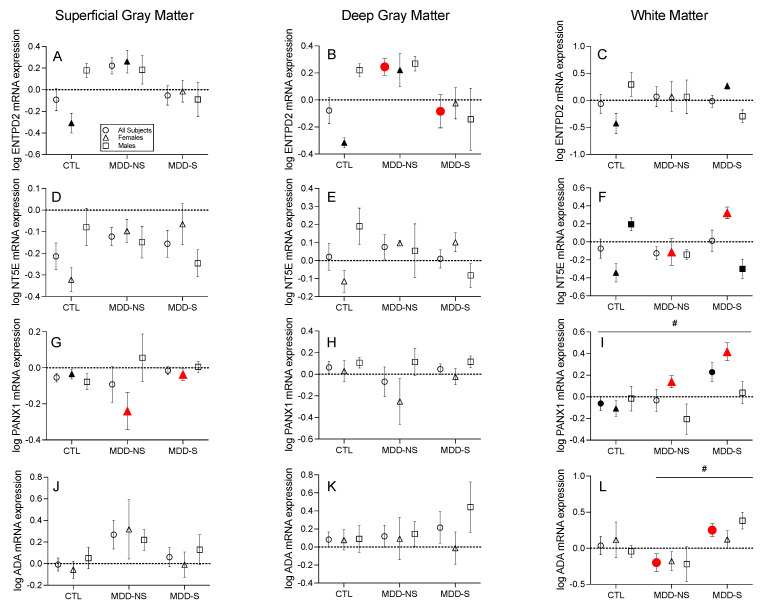
Purinergic Expression Changes among Extracellular ATP Catabolic Transcripts. (**B**) ENTPD2 mRNA expression was significantly lower in the MDD-S vs. MDD-NS all subjects group in the deep gray matter. (**F**) *NT5E* mRNA expression was significantly greater in the MDD-S vs. MDD-NS female group in the white matter. *PANX1* mRNA expression was significantly greater in the MDD-S vs. MDD-NS female group in the (**G**) superficial gray matter and (**I**) white matter. (**L**) *ADA* mRNA expression was significantly greater in the MDD-S vs. MDD-NS all subjects group in the white matter. Transcripts shown include those with at least one suicide-specific significant difference (i.e., between MDD-S and MDD-NS) in at least one comparison group (all subjects, females only, or males only) in at least one cell layer (superficial, deep, or white matter). If a suicide-specific significant difference was identified, data for that transcript are displayed across all cell layers. Of the displayed transcripts, (**A**–**C**) *ENTPD2*, (**F**) *NT5E*, and (**G**,**I**) *PANX1* exhibited additional significant differences between either MDD-NS or MDD-S compared to CTL subjects. These findings are discussed later in the context of Figure 3 but shown here for completeness. No significant differences between groups observed in (**D**,**E**,**H**,**J**,**K**). Points show the mean ± standard error of the mean (SEM) for all subjects (circles, *n* = 6–10), females only (triangles, *n* = 3–5), and males only (squares, *n* = 3–5). Filled symbols indicate significantly different transcript expression as determined by analysis of variance (ANOVA) and appropriate post hoc tests. Red shapes indicate suicide-specific significant differences and black shapes indicate non-suicide-specific significant differences. # indicates a significant main effect of disease in the all subjects comparison, determined by two-way ANOVA. Data and results of statistical tests are reported in Supplementary Tables S5 and S6. Abbreviations: CTL, control; MDD-NS, major depressive disorder—non-suicide; MDD-S, major depressive disorder—suicide; *ENTPD2*, ectonucleoside triphosphate diphospho-hydrolase-2; *NT5E*, ecto-5′-nucleotidase; *PANX1*, pannexin-1; *ADA*, adenosine deaminase.

*Extracellular ATP Catabolic Transcripts*: In the all subjects group, *ENTPD2* mRNA expression was significantly lower in the MDD-S compared to MDD-NS subjects in the deep gray matter (two-way ANOVA: F_(9,13)_ = 1.27 (sex effect), F_(2,13)_ = 3.61 (disease effect) *p* = 0.05, Figure 2B). In the female-only group, *NT5E* mRNA expression was significantly greater in the MDD-S compared to MDD-NS subjects in the white matter (one-way ANOVA: F_(2,10)_ = 13.37, *p* = 0.04, Figure 2F). *PANX1* mRNA expression was significantly greater in the MDD-S compared to MDD-NS female subjects in the superficial gray matter (one-way ANOVA: F_(2,10)_ = 4.66, *p* = 0.05, Figure 2G) and white matter (ANCOVA: F_(2,9)_ = 13.96, *p* = 0.03, after controlling for the effect of PMI, Figure 2I). *ADA* mRNA expression was significantly greater in the MDD-S compared to the MDD-NS all subjects group in the white matter (KW nonparametric test: KW statistic = 6.42, *p* = 0.04), with a significant main effect of disease observed (two-way ANOVA: F_(2,14)_ = 3.80, *p* = 0.05, Figure 2L).

### 2.2. Purinergic Transcript Expression Changes Common to Suicide and MDD Pathology

Here, we present significant differences between the MDD-S and CTL groups to highlight purinergic alterations that may be common to the pathophysiology of suicide and MDD.

**Figure 3:**
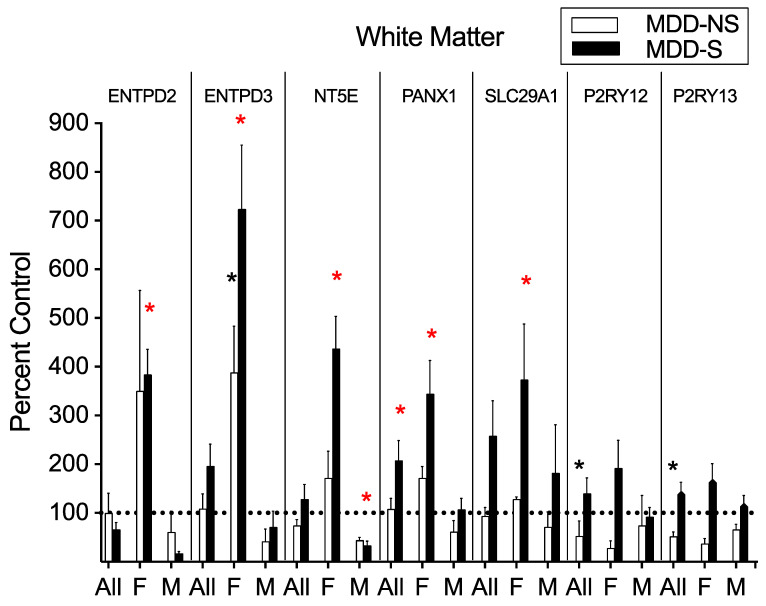
Purinergic Transcript Expression relative to Control groups in the White Matter. *ENTPD2*, *ENTPD3*, *NT5E*, *PANX1*, and *SLC29A1* mRNA expression was significantly greater in MDD-S vs. CTL females. *ENTPD3* mRNA expression was significantly greater in MDD-NS vs. CTL females. *NT5E* mRNA expression was significantly lower in the MDD-S vs. CTL males. *PANX1* mRNA expression was significantly greater in the MDD-S vs. CTL all subject group. *P2RY12* and *P2RY13* mRNA expression were significantly lower in the MDD-NS vs. CTL all subject groups. Data reported for MDD-NS (white bars) and MDD-S (black bars) groups as a percentage of the CTL group (dotted line showing mRNA expression for all control groups as 100%). A red star denotes a significant difference in mRNA expression for the MDD-S group relative to the CTL group. A black star denotes a significant difference in mRNA expression for the MDD-SS group relative to the CTL group. Transcripts displayed include those with at least one significant difference between MDD-NS or MDD-S compared to CTL groups in at least one comparison group (all subjects, females only, males only). *n* = 3–10 per group. Non-log transformed data were utilized to calculate percentages. Bar graph data presented as mean ± standard error of the mean (SEM). * *p* < 0.05. Data and results of statistical tests are reported in Supplementary Tables S5 and S6. Abbreviations: All, all subjects; F, female; M, male; MDD-NS, major depressive disorder—non-suicide; MDD-S, major depressive disorder—suicide; *ENTPD2*/*ENTPD3*, ectonucleoside triphosphate diphospho-hydrolase-2/3; *NT5E*, ecto-5′-nucleotidase; *PANX1*, pannexin-1; *SLC29A1*, equilibrative nucleoside transporter-1; *P2RY12*, purinergic receptor P2Y 12; *P2RY13*, purinergic receptor P2Y 13.

*Extracellular ATP Catabolic Transcripts*: Most significant alterations were observed in the white matter tissue isolated from female subjects. *ENTPD2* (one-way ANOVA: F_(2,10)_ = 5.20, *p* = 0.03), *ENTPD3* (one-way ANOVA: F_(2,10)_ = 28.99, *p* < 0.0001), *NT5E* (one-way ANOVA: F_(2,10)_ = 13.37, *p* = 0.03), *PANX1* (ANCOVA: F_(2,9)_ = 13.96, *p* = 0.004), and *SLC29A1* (ANCOVA: F_(2,9)_ = 7.15, *p* = 0.01, after controlling for the effect of PMI) mRNA expression was significantly elevated in the MDD-S compared to CTL female groups in the white matter (Figure 3). *NT5E* mRNA expression was significantly lower in the MDD-S compared to CTL males in the white matter (one-way ANOVA: F_(2,10)_ = 8.90, *p* = 0.01, Figure 3). In the all subjects analysis, *PANX1* mRNA expression in the white matter was significantly higher in the MDD-S compared to CTL subjects (ANCOVA: F_(2,22)_ = 5.86, *p* = 0.02, after controlling for the effect of PMI, Figure 3), with a significant main effect of disease observed (two-way ANOVA: F_(2,14)_ = 4.96, *p* = 0.02, Figure 1I).

*Purinergic Receptor Transcripts*: *P2RY13* was the only purinergic receptor transcript significantly elevated in the MDD-S compared to the CTL group, specifically among females in the superficial gray matter (one-way ANOVA: F_(2,10)_ = 7.11, *p* = 0.02, Figure S2).

### 2.3. Purinergic Transcript Expression Changes Exclusive to MDD Pathology

Here, we report significant differences found between the MDD-NS and CTL groups to highlight purinergic changes that are specific to the pathophysiology of MDD.

*Extracellular ATP Catabolic Transcripts*: In the white matter, *ENTPD3* mRNA expression was significantly greater in the MDD-NS compared to the CTL female group (one-way ANOVA: F_(2,10)_ = 28.99, *p* = 0.003, Figure 3). *ENTPD2* mRNA expression was significantly greater in the MDD-NS compared to CTL females in the superficial (one-way ANOVA: F_(2,10)_ = 7.30, *p* = 0.01) and deep (one-way ANOVA: F_(2,10)_ = 7.37, *p* = 0.01) gray matter (Figure S2). *PANX1* mRNA expression was significantly lower in the MDD-NS compared to CTL females in the superficial gray matter (one-way ANOVA: F_(2,10)_ = 4.66, *p* = 0.05, Figure S2).

*Purinergic Receptor Transcripts*: In the all subject analysis, *P2RY12* mRNA expression was significantly lower in the MDD-NS compared to CTL subjects in the white matter, with a significant main effect of disease observed (two-way ANOVA: F_(2,14)_ = 5.82, *p* = 0.01, Figure 2F and Figure 3), as well as deep gray matter (one-way ANOVA: F_(2,22)_ = 3.69, *p* = 0.04, Figure S2). *P2RY13* mRNA expression was also significantly lower in the MDD-NS compared to CTL subjects independent of sex in the white matter, with a significant main effect of disease observed (two-way ANOVA: F_(2,14)_ = 6.54, *p* = 0.01, Figure 2I and Figure 3).

### 2.4. Medication Analysis

**Figure 4:**
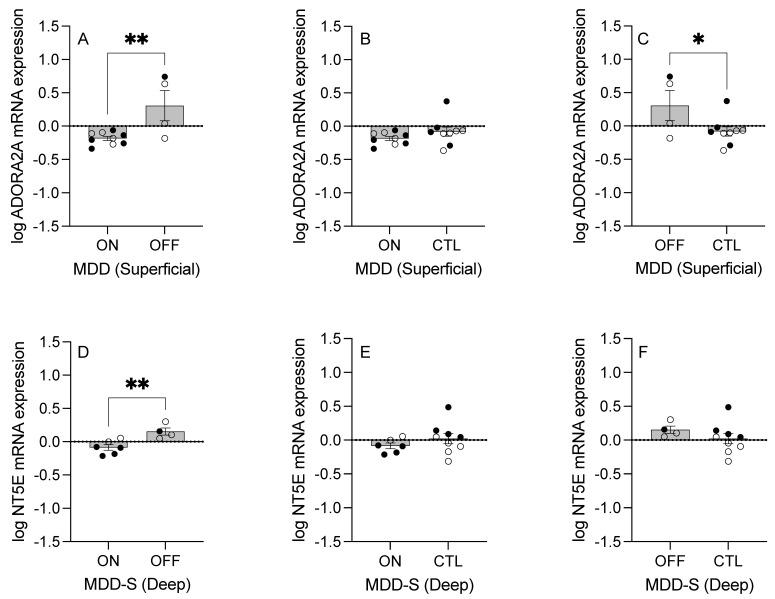
Medication Analysis—Significant mRNA Hits Across Cell Layers and Disease States. In the superficial gray matter tissue of MDD (non-suicide and suicide combined) subjects, (**A**) *ADORA2A* mRNA expression levels were significantly lower in MDD subjects on antidepressant medication compared to those off medication, while (**B**) no significant differences were observed in MDD subjects on medication vs. CTL subjects (*p* > 0.05), and (**C**) *ADORA2A* mRNA levels were significantly greater in MDD subjects off medication compared to CTL subjects. In the deep gray matter tissue of MDD-S subjects only, (**D**) *NT5E* mRNA expression levels were significantly lower in MDD-S subjects on antidepressant medication compared to those of medication. *NT5E* mRNA levels were not significantly different in MDD-S subjects (**E**) between subjects on medication and CTL groups or (**F**) between subjects off medication and CTL groups (*p* > 0.05). *n* = 4–9 per group. Outliers (*n* = 2) were removed from the *ADORA2A* on medication group based on the ROUT method (Q = 0.1%). Open circles indicate females, closed circles indicate males. Data presented as mean ± standard error of the mean (SEM). * *p* < 0.05. ** *p* < 0.01. Data and results of statistical tests are reported in Supplementary Table S7. Abbreviations: CTL, control; MDD, major depressive disorder; MDD-S, major depressive disorder—suicide; *ADORA2A*, adenosine A2A receptor; *NT5E*, ecto-5′-nucleotidase.

In an assessment of the effect of antidepressant medication on the purinergic transcript expression in MDD-NS and MDD-S subjects combined (MDD) in the superficial gray matter, *ADORA2A* mRNA expression was significantly greater in MDD subjects off compared to on medication (Welch’s-corrected t = 2.16, *p* = 0.001, Figure 4A). No significant differences were observed in *ADORA2A* expression in medication vs. CTL groups (Mann–Whitney U = 24, *p* > 0.05, Figure 4B). Significantly greater *ADORA2A* levels were observed in MDD subjects off medication compared to CTL subjects (t_(11)_ = 2.21, *p* = 0.05, Figure 4C).

In suicide (MDD-S) subjects only in the deep gray matter, *NT5E* was expressed at lower levels in subjects on compared to off medication (t_(8)_ = 3.54, *p* = 0.008, Figure 4D), however, no significant differences were found in *NT5E* levels between on medication and CTL groups (t_(13)_ = 1.09, *p* > 0.05, Figure 4E) or off medication and CTL groups (t_(11)_ = 1.10, *p* > 0.05, Figure 4F).

### 2.5. Results from Mapping RNA Expression Profiles to Cerebral Cortex Cell Types

Established expression data (Table S3) from the Human Protein Atlas’s Single Cell Type resource revealed the following purinergic gene-to-most abundant cerebral cortex cell type relationships: *ADA*: pericytes; *ADORA1*: oligodendrocytes; *ADORA2A*: pericytes; *ALPL*: endothelial cell; *ENPP2*: oligodendrocyte; *ENTPD1*: CNS macrophage; *ENTPD2*: astrocyte; *ENTPD3*: interneuron; *NT5E*: fibroblast; *P2RX4*: CNS macrophage; *P2RX7*: committed oligodendrocyte precursor cell; *P2RY12*: CNS macrophage; *P2RY13*: CNS macrophage; *P2RY14*: pericyte; *SLC29A1*: interneuron; *PANX1*: CNS macrophage.

## 3. Discussion

In the present exploratory study, we identified components of the purinergic system—receptors, enzymes, channels, and transporters—involved in adenosine metabolism that have altered gene expression in the white matter as well as the superficial and deep gray matter cell layers from hard to obtain human postmortem DLPFC tissue in suicide and MDD. While preliminary, these results expand upon prior literature that implicates the purinergic system in severe mental illnesses and suggest extensive sex- and cell-layer-specific transcript expression dysregulation of this system in suicide and MDD.

### 3.1. Purinergic System Dysregulation in Suicidality—A Focus on Receptors

To identify purinergic transcript changes that may be specific to the pathophysiology of suicide, we analyzed expression levels between the MDD-S and MDD-NS groups. These comparisons revealed several alterations, primarily among purinergic receptors in the DLPFC white matter—a cell layer abundant in oligodendrocytes and astrocytes [39]. Alongside components that regulate the ATP-to-adenosine ratio, purinergic receptors are crucial as they mediate the pathway’s downstream effects. In our white matter analysis, we observed a significant elevation in the transcript expression of *P2RX4*, *P2RY12*, and *P2RY13* in the MDD-S subject group regardless of sex. These results contribute to the postmortem and transcriptomic findings in suicide published over the past four years.

In 2020, Zhang et al. conducted a postmortem study focusing on the anterior cingulate cortex (ACC) of subjects with schizophrenia who died by suicide and found that these individuals exhibited a 1.5-fold increase in *P2RY12* mRNA expression compared to non-suicide and healthy control subjects [40]. Consistent with this finding, Naggan et al. conducted a similar postmortem analysis of postmortem hippocampal tissue and observed a significant increase in *P2RY12*-labeled microglia density in bipolar disorder patients who had died by suicide compared to non-suicide and control subjects [41]. The DLPFC was also examined in these studies with no differences detected, partially attributed to the relatively small sample volumes. However, in a larger postmortem transcriptomic analysis utilizing RNA sequencing from the DLPFC in individuals who died by suicide, Punzi et al. discovered that *P2RY12*, *P2RY13*, and a *P2RY14* paralog, *GPR34*, were among the top differentially expressed genes (DEG) in individuals who died specifically by violent suicide (i.e., firearms, hanging) [31]. Although traditionally known for their involvement in immune cell signaling [42], the enriched microglia-associated purinergic P2Y receptor signaling in these DEG profiles also shows similarities to a genome-wide association study (GWAS) of aggressive phenotypes in *Drosophila* [43]. It follows that P2Y-mediated signaling in the brain may facilitate the transition from suicidal ideation to violent suicidal behavior, predominantly observed in males.

Male-specific purinergic alterations in our study uncovered elevated *P2RY13* mRNA expression in the suicide compared to the non-suicide group in the white matter, where 80% of males died by violent means. However, we observed similar increases in female-specific groups in the white matter and superficial gray matter—a cell layer rich in neuronal cell bodies and astrocytes [44,45]—despite all female suicides being due to overdose (i.e., non-violent means). This suggests that *P2RY13* alterations may not be exclusively associated with violent suicide and that aggressive behavior could play a role in suicide capability, regardless of the method employed [46]. In conjunction with the Zhang and Punzi studies, our results substantiate the theory that regardless of underlying psychiatric diagnoses, suicide is a phenotype biologically distinct and separable by alterations in purinergic signaling.

Our study’s identification of elevated *P2RX4* expression is a key finding, as this receptor has not been extensively studied in MDD or mood disorders. In contrast, the *P2RX7* receptor has been implicated in depressive-like and specifically suicidal behavior through its role in inducing pro-inflammatory cytokines like interleukin-1β (IL-1β) [47]. Elevated IL-1β levels have been observed in the brain and blood of individuals who have died by suicide or attempted suicide [48,49]. Animal studies further demonstrate that hypothalamic and midbrain injections of IL-1β increase aggressive behavior, suggesting that *P2RX7*-mediated IL-1β production may contribute to aggression and suicidal tendencies [50,51]. Our findings reveal significant differences in *P2RX4* gene expression, but not *P2RX7*, suggesting that purinergic signaling abnormalities in suicide may extend beyond the previously recognized P2Y receptor family and microglial involvement. This warrants further investigation into *P2RX4* in the pathophysiology of suicide.

### 3.2. Purinergic System Dysregulation in Suicidality—Extracellular Enzymes, Channels, and Transporters

When comparing the MDD-S and MDD-NS groups, we also identified novel suicide-specific alterations in transcripts involved in extracellular ATP catabolism. The regulation of extracellular adenosine and ATP levels is a cell-specific process. Neurons, glia, and immune cells release ATP through distinct mechanisms such as vesicular exocytosis and PANX1 channel-mediated pathways [52]. Once in the extracellular space, ATP is rapidly catabolized to adenosine via the sequentially hydrolyzing ectonucleotidases (ENTPDs and NT5E), a process more prevalent in glial cells [53]. Alternatively, adenosine may be directly released from neuronal cells via ENTs, a process more prevalent in neurons [54]. Further cell-specific studies are warranted to continue to understand the relative contributions of indirect vs. direct generation of adenosine in the brain; however, in the pathological condition of suicide, our results suggest that cell-layer-specific mechanisms contribute to extracellular adenosine dysregulation.

*ENTPD2*, primarily expressed in astrocytes [55], hydrolyzes ATP to ADP and AMP and modulates purinergic signaling by controlling nucleotide availability [56]. Extracellular ATP acts as a danger signal, activating immune responses and promoting inflammation through purinergic P2 receptors on immune cells [52]. Conversely, adenosine exerts anti-inflammatory effects via P1 (adenosine) receptors to protect neural tissue [57]. This balance between ATP and adenosine is vital in controlling immune cell activation and migration [58], essential processes for injury or infection response. In our analysis, *ENTPD2* gene expression levels were significantly lower among the suicide subjects in the deep gray matter—a cell layer rich in various neuron types and astrocytes [44,45]. Reduced *ENTPD* levels may affect the amount of substrate available for conversion to adenosine, affecting purinergic and adenosine receptor activation and downstream neuroprotection [59].

ADA is another enzyme in the catabolic cascade that provides a means of inactivating adenosine by degrading it to inosine, reducing its anti-inflammatory effects [60]. In the white matter, we observed significantly elevated *ADA* transcript levels among suicide subjects. Pathologically elevated ADA activity may contribute to decreased adenosine availability, diminishing its protective roles in the brain, as well as higher uric acid, the end product of purine metabolism [61]. While excessive uric acid is linked to self-injurious behaviors, as is seen in Lesch–Nyhan syndrome [62], recent studies show an association between lower serum uric acid levels and increased suicide risk in individuals with MDD [63,64]. This suggests a complex relationship between uric acid and neuropsychiatric conditions, especially given that allopurinol, which reduces uric acid levels, has been explored as a therapeutic for mania [65]. Although further postmortem studies are needed to explain the precise mechanisms linking ADA, uric acid, and suicide risk, our findings align with observed white matter abnormalities in MDD and suggest a role for increased *ADA* in exacerbating white matter integrity problems known to positively correlate with increased illness severity [66].

Female-specific changes were also observed, particularly involving *PANX1* and *NT5E*. *PANX1* encodes Pannexin-1, a membrane channel that facilitates the extracellular release of ATP and influences neuroinflammatory processes [67]. Significantly elevated *PANX1* transcript levels were detected in the superficial gray matter and white matter of female suicide subjects compared to non-suicide counterparts. This upregulation may contribute to dysregulated ATP signaling and neuroinflammation, factors implicated in mood disorders [68], which are generally more prevalent in females. However, this finding contrasts with the global gender paradox in suicide rates, where males have a higher incidence than females [69]. *NT5E* transcripts, encoding the enzyme CD73 that hydrolyzes AMP to adenosine [70], were also significantly more abundant in the white matter of female suicide subjects. This aligns with studies reporting elevated *NT5E* levels in the hippocampus of stress-induced mouse models of depression, where inhibition of *NT5E* ameliorated depressive phenotypes [71].

### 3.3. Shared Purinergic System Alterations in MDD and Suicide

To explore purinergic transcript alterations that may be common to the pathophysiology of MDD and suicide, we compared expression levels between MDD-S and CTL groups. Several significant changes between these groups emerged among the extracellular enzymes, channels, and transporters, notably within the DLPFC white matter isolated from female subjects.

The significant elevation of *ENTPD2*, *ENTPD3*, *NT5E*, *PANX1*, and *SLC29A1* transcripts observed in the white matter of females with MDD who died by suicide highlights a potential sex-specific purinergic dysregulation in MDD that coincides with suicidality. ENTPD3, like ENTPD2, hydrolyzes ATP to ADP and AMP but is predominantly expressed in neurons [55]. Elevated *ENTPD* and *NT5E* levels suggest complications with ATP signaling, which promotes neuronal activity, synaptic plasticity, and repair under physiological conditions [59]. Excessive ATP hydrolysis may represent either a compensatory effect or a mechanism that suppresses baseline functions, reducing neuroprotective signaling in the DLPFC [72]. Given that our study examines mRNA expression at a single postmortem time point, this interpretation remains speculative. However, such dysregulation could contribute to behavioral deficits associated with this brain region such as executive dysfunction and a reduced capacity to manage stress [73].

In the context of elevated *PANX1* in females, ATP hydrolysis may be a response to elevated ATP availability. Notably, elevated *PANX1* levels were also observed among MDD-S subjects regardless of sex, indicating that *PANX1* changes may not be exclusive to females. PANX1 channels are the principal conduits of ATP release in the brain, initiating a complex inflammatory cascade, including immune cell infiltration and inflammasome activation [67]. This environment may necessitate rapid ATP degradation to shift the ratio toward adenosine. Adenosine, in the absence of ADA activity, activates A_1_ receptors to act as a potent anti-inflammatory and immunosuppressive agent [57,74].

While ENTPD-driven reduction of inflammation is typically protective, the additional finding of elevated *SLC29A1* levels in females indicates that adenosine may be transported intracellularly, leading to a hypoadenosinergic state [75], which is pathological in other neuropsychiatric illnesses such as schizophrenia [76,77,78,79]. ENT1, encoded by *SLC29A1*, is considered the primary equilibrative nucleoside transporter subtype in neuronal and glial cells across the gray and white matter of the frontal cortex [55,80]. Given the parallel upregulation of ectonucleotidases and *PANX1* in females and the bidirectional nature of ENTs, *SLC29A1* could exacerbate or mitigate extracellular ATP-to-adenosine imbalances. This imbalance may intensify an inflammatory environment by limiting adenosine’s ability to counteract inflammation [57].

Among males, *NT5E* transcript expression was significantly greater in controls compared to the suicide group in the white matter. At basal levels, males may possess a robust ability to balance the extracellular ATP to adenosine ratio, similar to *Nt5e* knockout male mice compensating with tissue-nonspecific alkaline phosphatase (*Alpl*) to maintain adenosine production and overall neuroprotection [81]. *Nt5e* knockout female mice lack this baseline compensation [81], and thus may only upregulate extracellular enzymes under conditions of extreme stress (e.g., suicide), suggesting a delayed compensatory response. Notably, *ALPL* transcripts, abundantly expressed in brain endothelial cells [55], did not show significant alterations in our study.

Among the purinergic receptors, *P2RY13* transcripts were uniquely elevated in the superficial gray matter of the female suicide group. While also observed in the suicide-specific purinergic analysis, this finding suggests that perturbations in *P2RY13* may represent a shared mechanism underlying MDD and suicide.

### 3.4. MDD-Specific Purinergic System Dysfunction

To best decipher purinergic transcript alterations that may be exclusive to MDD pathophysiology, we compared gene expression levels between MDD-NS and CTL groups. Fewer findings emerged than the previous comparisons and most were among the purinergic receptors.

In the context of MDD, purinergic A_2A_ receptors as well as P2RX7 receptor antagonism have shown the most positive results in terms of antidepressant effects, especially relative to the P2Y receptor family [82,83]; however, neither of these transcripts was significantly altered in our comparisons. We observed *P2RY12* transcript levels to be significantly lower in non-suicide subjects within the deep gray matter and white matter, while *P2RY13* transcripts were significantly reduced exclusively in the white matter of these subjects. Our results are in line with postmortem findings from Zhang et al., who reported a significant decrease in *P2RY12* mRNA expression in the ACC of MDD-NS individuals compared to healthy controls [30]. While P2Y receptors are increasingly noted for their involvement in depressive states [84], the primary focus remains on their neuroinflammatory roles in the microglial activity that is heavily associated with suicide; thus, our findings warrant functional studies on P2Y involvement specifically in MDD.

In contrast to the shared MDD and suicide finding of significantly elevated *ENTPD2* and *PANX1* transcripts in the white matter isolated from females, here we discovered significantly reduced *PANX1* in the context of significantly elevated *ENTPD2* transcript levels in the superficial gray matter of the MDD-NS female group. Interestingly, the absence or blockade of *Panx1* in the adult mouse brain has been shown to increase synaptic transmission and maintain learning, suggesting that compensatory mechanisms stabilize their neuronal activity [85]. In the chronic social defeat stress mouse model, elevated *Entpd* expression leads to depression-like behaviors that are only reversed with *Entpd* knockdown or antagonism [86]. These findings collectively suggest the involvement of perturbed adenosine signaling, which may be more prominent in females. Whether adenosine levels are increased in the brains of individuals with MDD remains an open question requiring further investigation.

### 3.5. Cell Layer Differences

To enhance the resolution of purinergic transcript detection, we isolated the superficial and deep gray matter as well as the white matter DLPFC cortical layers using LMD. With this approach, we captured more precise gene profiles that may not have been possible with mixed cell types and layers in whole brain homogenate, reflecting the distinct physiological roles of each layer.

Approximately 70% of the purinergic alterations identified in this study were localized to the DLPFC white matter, a cell layer that is enriched with oligodendrocytes and astrocytes [39] and is critical for the rapid transmission of information across the brain via corticocortical connectivity [36]. Our results align with postmortem and neuroimaging studies that associate white matter abnormalities in individuals who have died by suicide. For instance, cell counts of microglia—the CNS’s resident macrophages—are elevated in the prefrontal white matter of those who have died by suicide, suggesting an inflammatory response contributing to suicide pathology [87]. Supporting this, neuroimaging studies in individuals with MDD who have contemplated or attempted suicide reveal disrupted white matter structural networks, characterized by decreased local efficiency and increased path length, which reflect impaired communication pathways underlying cognitive and emotional deficits in suicidal ideation [88]. Elevated pro-inflammatory cytokines detected in the white matter of suicide victims may exacerbate these disruptions, further compromising white matter connectivity [89].

About 30% of the purinergic changes identified in this study were localized to the superficial and deep layers of the DLPFC gray matter. Like the white matter, these layers are enriched with astrocytes, though the deep layers have a higher density of glial cells, while both layers primarily contain various neuronal cell bodies [45]. Functionally, the superficial layers are involved in higher-order processes such as sensory perception and voluntary movement through communication with other cortical and subcortical regions, whereas the deep layers relay information by projecting primarily to subcortical brain structures [37]. Our findings indicate that while purinergic alterations are predominantly concentrated in the white matter in suicide, the gray matter layers also contribute.

Our mapping of purinergic RNA expression profiles to cerebral cortex cell types further helped in contextualizing our findings. This analysis showed that many of the alterations among purinergic transcripts occurred in cell layers consistent with known expression patterns. For example, *ADA*, which is predominantly expressed in pericytes but also found in oligodendrocytes and, to a lesser extent, astrocytes [55], shows its highest enzymatic activity in the white matter of the frontal lobe [90]—aligning with the cell layer where we observed significant *ADA* transcript changes. Similarly, the purinergic P2 receptors significantly altered in our study (*P2RX4*, *P2RY12*, *P2RY13*), which are abundantly expressed on microglia, a subtype of CNS macrophages, in gray and white matter [55], showed prominent dysfunction in the white matter, with additional changes observed in the superficial and deep gray matter layers. *ENTPD2*, a gene highly expressed in astrocytes [55], showed significant perturbations across all layers examined in our analysis, which aligns with the fact that their RNA expression is ubiquitous across cell layers.

These findings overall emphasize a complex spatial distribution of purinergic dysregulation, where the distinct roles of cell layers and cell types within those layers shape the observed patterns in suicide pathology.

### 3.6. Medication Effects on Purinergic Transcripts

When evaluating the impact of antidepressants on purinergic gene expression, our analysis revealed that *ADORA2A* levels were significantly reduced in MDD subjects on antidepressants compared to those off-medication, but significantly greater in the off-medication group relative to controls in the superficial gray matter. This suggests that elevated *ADORA2A* levels in MDD may be normalized by chronic antidepressant treatment. While our study did not find elevated *ADORA2A* levels in the DLPFC of individuals with MDD, research using the chronic unpredictable mild stress mouse model has demonstrated an association between increased A_2A_ receptors in the striatum with depression-like behaviors [91]. Further, our medication findings mirror prior research on antipsychotics’ effects on *ADORA2A* in schizophrenia [78], indicating a shared medication mechanism across neuropsychiatric illnesses implicating mood imbalances.

We also observed that *NT5E* levels were significantly lower in MDD-S subjects on antidepressants compared to those off-medication in the deep gray matter, with no significant differences observed between on- or off-medication groups and controls. Our study observed significantly elevated *NT5E* levels in the white matter DLPFC tissue isolated from female MDD-S subjects compared to their MDD-NS as well as control counterparts, but this was exclusive to the white matter. Thus, our medication finding may suggest that (I) while medication reduces *NT5E* levels in suicide-prone MDD individuals, it may not fully normalize them, and (II) downregulation of *NT5E* via antidepressants may potentially offer therapeutic benefit in a cell layer-dependent manner.

### 3.7. Limitations and Future Directions

The preliminary data presented provide novel, sensitive, and quantitative information on the transcript expression of purinergic components in suicide and MDD, however, some limitations should be considered. First, although we were sufficiently statistically powered for comparisons between all subjects, the sample sizes for the sex-specific and medication analyses were relatively small (Supplementary Methods), limiting the generalizability of our findings and necessitating replication in larger cohorts. Studying postmortem tissue also prevents causal inferences regarding whether observed changes were a consequence of MDD or suicide or if they contributed to their onset; thus longitudinal in vivo studies are essential to further delineate causal relationships. In the medication analysis, relatively few subjects were not taking antidepressant medication at the time of death; thus, the impact of medication on purinergic transcript expression needs to be validated in larger studies that consider different classes of antidepressants as well as the effects of other over-the-counter medications that may impact the expression of purinergic system components. While we focused on the DLPFC, purinergic dysregulation may extend to other brain regions involved in mood disorders and decision-making, such as the hippocampus and amygdala, which were not examined. Finally, transcript-level changes do not always correlate to protein expression or function. Future studies should aim to validate our findings at the protein level in larger sample sizes across a broader range of implicated brain regions to further understand the purinergic system in suicidality. Conducting analyses in which subjects are stratified by the method of death (e.g., violent vs. non-violent suicide), where possible, as well as sex may also help to reduce some of the heterogeneity implicit in suicide and advance our understanding of the underlying molecular biology. Future work should also build upon the present transcript-level findings by conducting in-depth analyses of the spatiotemporal dynamics of ATP and adenosine in the brain [72] to fully appreciate the complexities of purinergic signaling in neuropsychiatric illnesses.

## 4. Materials and Methods

### 4.1. Subjects

Postmortem human brain tissue sourced from the DLPFC brain region was obtained from the Maryland Brain Collection, with the proper consent from the next of kin following IRB-approved protocols. The samples included non-psychiatrically ill subjects as controls (CTL, *n* = 9–10), those diagnosed with MDD who did not die by suicide (MDD-NS, *n* = 6), and those diagnosed with MDD who died by suicide (MDD-S, *n* = 9). Sample size explanations are provided in the Supplementary Methods. The groups were matched for sex, age, postmortem interval (PMI), and pH (log base 10 of the hydrogen ion concentration) if provided (Table 1). Immediately after dissection, the samples were frozen and preserved at −80 °C for further experiments. Diagnoses for patients prior to death were confirmed by two psychiatrists independently through reviews of medical records, autopsy reports, and family interviews, which followed the Structured Clinical Interview for the Diagnostic and Statistical Manual of Mental Disorders, Fourth Edition (DSM-IV). The medication status was classified as “on” for subjects who had been consuming antidepressant medication regularly within the last six weeks of life prior to death. The type of antidepressant patients had been consuming was derived from toxicology reports. Comprehensive demographic data are reported in the Supplementary Table S1.

### 4.2. Laser Microdissection (LMD)

The Leica Laser Microdissection 6 instrument (Leica Microsystems, Wood Dale, IL, USA) was utilized to perform LMD and excise Nissl-stained tissue sections from the superficial (layers II–III) and deep (layers V–VI) gray matter as well as the white matter of the DLPFC. The LMD procedures followed previously established methods [76,92,93,94]. Briefly, frozen tissue sections (14 µm) were thawed at room temperature, rehydrated with distilled water, and Nissl stained using an RNAse-free cresyl violet solution (1% cresyl violet, 1% glacial acetic acid, pH 4.0) (FD NeuroTechnologies, Columbia, MD, USA) to highlight neuronal cell bodies and differentiate gray and white matter. After ethanol washes and histoclear treatment, cell layers were identified by their morphology and neuronal density and excised from the DLPFC using LMD. Histologically, layers II–III were characterized by their smaller, tightly packed pyramidal neurons, layers V–VI by their larger pyramidal neurons in a more dispersed organization compared to layers II–III, and white matter by its relatively less cellular environment and abundance of oligodendrocytes and myelinated axons, lacking the neuronal profiles seen in gray matter [95,96]. LMD was performed under a 40x objective lens with these laser settings: power: 24–25, aperture: 4–5, and speed: 8. The cell layers were collected into the caps of 0.5 mL tubes (Axygen, Union City, CA, USA) for each subject, incubated with 30 μL of PicoPure RNA extraction buffer (Applied Biosystems, Foster City, CA, USA) for 30 min at 42 °C, centrifuged for 2 min at 400× *g*, and stored at −80 °C until further experiments.

### 4.3. RNA Extraction, Complementary DNA (cDNA) Synthesis, and cDNA Pre-Amplification

The PicoPure RNA isolation kit (Molecular Devices, Sunnyvale, CA, USA) was used to extract RNA from cell layer-specific postmortem tissue samples according to the manufacturer’s instructions. Subsequently, the High-Capacity cDNA Reverse Transcription Kit (Applied Biosystems, Foster City, CA, USA) was used to synthesize cDNA using 10 μL of total RNA. TaqMan primers (*n* = 16) (ThermoFisher Scientific, Waltham, MA, USA) for the purinergic components of interest as well as for housekeeping genes glyceraldehyde-3-phosphate dehydrogenase (*GAPDH*), beta2-microglobulin (*B2M*), beta actin (*ACTB*), and cyclophilin A (*PPIA*) (*n* = 4) were utilized (Table S2). Primers were pooled for the pre-amplification polymerase chain reaction (PCR), diluted with RNase/DNase-free water to a final concentration of 0.2×, and mixed with cDNA and FastStart Universal Mastermix (Roche Life Sciences, Indianapolis, IN, USA). The PCR protocol was as follows: an initial denaturation step at 95 °C for 10 min followed by 14 cycles of denaturation at 95 °C for 14 s and annealing at 60 °C for 4 min. After pre-amplification, the samples were diluted 1:3 with RNase-free water and stored at −20 °C until further use in real-time quantitative PCR assays.

### 4.4. Quantitative Polymerase Chain Reaction (qPCR) Assays

qPCR assays were performed for each subject in triplicate using 384-well optical reaction plates (Life Technologies, Carlsbad, CA, USA) with all subjects randomized by disease and sex. The experiment design is outlined in the Supplement (Figure S1). qPCR plates were analyzed using an Applied Biosystems detection system (ABI SteponePlus, Life Technologies, Carlsbad, CA, USA). Each reaction included 0.5 μL of pre-amplified cDNA in a 10 μL mixture containing 9.5 μL of mastermix and a 1× concentration of each primer (Applied Biosystems, Life Technologies, Carlsbad, CA, USA). Negative controls included reactions without cDNA (non-template control) and reactions without reverse transcriptase (no RT control). Once plated, the reaction began with an initial ramp time of 10 min at 95 °C, followed by 40 cycles of 15 s at 95 °C, and 1 min at 60 °C for annealing. The relative concentrations of the transcripts were calculated independently for primers and cell layers using a standard curve generated from cDNA dilutions pooled from all subjects. Transcript levels were normalized to the geometric mean of the four reference genes *GAPDH*, *B2M*, *ACTB*, and *PPIA*, which showed consistent expression across the CTL, MDD-NS, and MDD-S groups in all comparisons of interest (analysis of variance (ANOVA) test, *p* > 0.05).

### 4.5. Mapping RNA Expression Profiles to Cerebral Cortex Cell Types

To contextualize our findings, we analyzed RNA expression profiles of purinergic system transcripts across specific cerebral cortex cell types, comparing established expression patterns with our study results (Table S3). This analysis utilized data from the Human Protein Atlas’s Single Cell Type resource, which integrates single-cell RNA sequencing (scRNAseq) and deconvolution of bulk transcriptomics to provide comprehensive insights into gene expression across various human tissues and cell types [55].

### 4.6. Data Analysis

For each purinergic transcript, data were tested for normal distribution using the D’Agostino and Pearson omnibus normality test. The datasets were not normally distributed for any of the transcripts, so they were log-transformed. Each transcript’s log-transformed dataset was re-tested for normal distribution using the D’Agostino and Pearson omnibus normality test and subsequently for homogeneity of variance using either the Brown–Forsythe (for non-normally distributed data) or Bartlett test (for normally distributed data). For visual comparisons to the control group (Figure 3, Figure S2), non-log-transformed data were utilized to calculate percentages.

Our primary analysis pooled data from females and males, and our secondary analysis assessed females and males separately. To ensure the validity of our regression analyses, residual analyses were conducted before regression modeling to assess associations between transcript expression and age and PMI. For significant associations, an analysis of covariance (ANCOVA) test was conducted, and Bonferroni’s post hoc multiple comparison analysis was performed to assess significant pairwise comparisons.

In the absence of significant associations, one of the following tests was performed based on the comparison of interest (Table S4): a one-way ANOVA parametric test was used when data were normal and variances were equal, a Kruskal–Wallis nonparametric test was utilized when normality was violated, and Welch’s unequal variance test was selected when variance was unequal. To address multiple comparisons for significant findings and assess statistically significant pairwise comparisons, appropriate post hoc analyses were performed: Tukey analyses for parametric tests, Dunn’s analyses for nonparametric tests, and Dunnett’s T3 analyses for unequal variance tests.

To assess the effect of sex and diagnosis as biological variables, a two-way ANOVA was performed with sex as a between-subjects factor and diagnosis as a within-subjects factor. Since an interaction term could not be calculated, the two-way ANOVA allowed for sex and disease effects to be examined independently. A Tukey post hoc multiple comparison analysis was conducted to assess significant pairwise comparisons.

For pairwise comparisons in the medication analyses, data were tested independently for normal distribution using the D’Agostino and Pearson omnibus normality test and subsequently for homogeneity of variance using the F-test. Based on the results, one of the following tests was performed: unpaired two-tailed parametric Student’s *t*-test, Mann–Whitney nonparametric test, or Welch’s unequal variance test.

An alpha level of 0.05 was used for all statistical tests. Data were analyzed using GraphPad Prism version 10.2.3 (GraphPad Software, La Jolla, CA, USA) and Statistica version 13.0 (Statsoft, Tulsa, OK, USA).

## 5. Conclusions

In conclusion, the present exploratory postmortem analyses provide the most comprehensive information to date on purinergic transcript expression alterations in the DLPFC, stratified by cell layer, among suicide-prone individuals with underlying MDD. Distinct transcript alterations in this system, primarily white matter- and female-specific, provide a foundation for understanding how the purinergic metabolic pathway influences ATP catabolism and purinergic receptor dysregulation, potentially contributing to the underlying pathology of MDD and suicidality. Together, these data provide important new information necessary for future mechanistic studies on the contribution of the purinergic system’s role in CNS inflammatory processes that lead to neuropsychiatric illnesses and suicidality.

## Figures and Tables

**Table 1:** Subject Demographics.

	N	Sex	Age	PMI (Hours)	pH
**CTL (SF, D)**	9	5 F/4 M	36.4 ± 8.5 (23–49)	13.9 ± 4.4 (7–21)	6.6 ± 0.4 (6.1–7.4)
**CTL (W)**	10	5 F/5 M	38.2 ± 9.8 (23–54)	14.2 ± 4.3 (7–21)	6.6 ± −0.4 (6.1–7.4)
**MDD-NS**	6	3 F/3 M	49.3 ± 6.0 (42–58)	21.3 ± 3.6 (15–25)	6.2 ± −0.6 (5.8–7.1)
**MDD-S**	10	5 F/5 M	37.2 ± 6.0 (30–45)	17.2 ± 7.3 (7–31)	6.4 ± 0.2 (6.2–6.5)

Data for subjects utilized in the study. Postmortem dorsolateral prefrontal cortex (DLPFC) tissue obtained from the Maryland Brain Collection. Data presented as mean ± standard deviation. Data ranges are in parentheses. Abbreviations: CTL, control; MDD-NS, major depressive disorder—non-suicide; MDD-S, major depressive disorder—suicide; SF, superficial gray matter tissue; D, deep gray matter tissue; W, white matter tissue; N, number of subjects; F, female; M, male; PMI, postmortem interval; pH, log_10_[H+].

## Data Availability

Data are contained within the Article and Supplementary Materials. Any additional data from statistical analyses will be made available upon request.

## Supplementary Materials

The supporting information can be downloaded at: https://www.mdpi.com/article/10.3390/ijms26051826/s1. References [55,94,97,98,99] are cited in the Supplementary Materials.
