# Supplementary material for: Purinergic System Transcript Changes in the Dorsolateral Prefrontal Cortex in Suicide and Major Depressive Disorder
Source: Int J Mol Sci. 2025 Feb 20;26(5):1826. doi: 10.3390/ijms26051826 (PMC11898938; doi:10.3390/ijms26051826)
Supplement: Supplementary file 1 [file ijms-26-01826-s001.zip › ijms-3467447-supplementary.pdf]

Supplementary Materials.

Contents

Contents	
Supplementary Methods	Page 2
Supplementary Tables & Captions	Page 3
Supplementary Figures & Captions	Page 12
Supplementary References	Page 14

Supplementary Methods.

We performed a sample size calculation utilizing data from our previously published study (1) measuring mRNA expression of GLUT3 in SCZ vs WT postmortem dorsolateral prefrontal cortex (DLPFC) tissue as pilot data for our present study: Given $\alpha = 0.05$, and power $(1–\beta) = 0.8$, we utilized the following population values for our calculation: $\mu CTL = 0.71$ ($\sigma = 0.24$, Δ ($\mu CTL–\mu SCZ$) $= 0.39$. Given this information, $n = 6$ / group (2, 3).

We prioritized maintaining rigorous matching of subjects by age, sex, pH (where possible), and post-mortem interval (PMI). We avoided including subjects from different brain banks to maintain consistency in tissue processing and reduce variability that may have compromised the validity of our findings. While this approach strengthened the reliability of the tissue and data used, it limited the number of subjects in certain analyses, such as the sex and medication comparisons. Consequently, these analyses may have lacked sufficient statistical power to detect all existing effects.

We refrained from performing reverse power calculations on our significant findings due to several challenges with this method, such as logical invalidity and misleading interpretations (4). Post-hoc power is derived from observed data after the completion of a hypothesis test, treating certain variables as constants rather than random variables, violating the foundational concept of power. Also, the interpretation of post-hoc power effectively restates the result of the test rather than offering independent insight. Thus, we utilized a published study as pilot data to calculate sample size for the present study.

Supplementary Tables & Captions.

Table S1: Comprehensive Demographics of Subjects from the Maryland Brain Collection.

Subject #	Race	Sex	Diagnosis	Cause of Death	Medication	Antidepressant type	Age	pH	PMI
1	W	F	CTL	smoke inhalation, accidental	off	N/A	23	6.54	12
2	W	F	CTL	cardiac arrhythmia, dissection of coronary artery	off	N/A	31	6.66	21
**3**	W	M	CTL	atherosclerotic cardiovascular disease (ASCVD)	off	N/A	54	6.9	17
4	W	M	CTL	manner undetermined, narcotic intoxication	off	N/A	45	6.1	17
5	W	F	CTL	deep vein thrombosis due to leg fracture	off	N/A	37	6.49	10
6	W	M	CTL	electrocution	off	N/A	28	6.3	13
7	B	M	CTL		off	N/A	49	6.76	19
8	B	M	CTL	Hypertensive ASCVD	off	N/A	41	6.68	14
9	W	F	CTL	dilated cardiomyopathy, opioid abuse	off	N/A	42	6.21	12
10	W	F	CTL	cardiac arrhythmia, 7 days post-partum septic	off	N/A	32	7.37	7
11	W	F	MDD-NS	ASCVD, mixed drug intoxication	on	Wellbutrin	46	7.05	23
12	W	M	MDD-NS	ASCVD, complicating hyperthermia		N/A	58	6	24
13	W	F	MDD-NS	diabetic ketoacidosis	on	Fluoxetine	47	5.8	25
14	B	F	MDD-NS	ASCVD	on	Fluoxetine	55		20
15	W	M	MDD-NS	manner undetermined, cocaine and narcotic intoxication	on	Fluoxetine	48		15
16	W	M	MDD-NS	drowning, accidental	on	Cyclobenzaprine	42	6.1	21
17	W	F	MDD-S	suicide, overdose	off	N/A	40	6.51	20
18	W	M	MDD-S	suicide, hanging	on	Doxepin	43	6.31	20
19	W	M	MDD-S	suicide, hanging	on	Metabolite of Venlafaxine	30		17
20	W	M	MDD-S	suicide, narcotic and acetaminophen intoxication	off	N/A	43		12
21	W	F	MDD-S	suicide, narcotic intoxication	off	N/A	41		7
22	W	M	MDD-S	suicide, gunshot wound (GSW) to chest	on	Nortriptyline	37		11
23	W	F	MDD-S	suicide, multiple drug intoxication	off	N/A	45	6.49	31
24	W	F	MDD-S	suicide, nortriptyline intoxication	on	Nortriptyline	30	6.18	16
25	W	F	MDD-S	suicide, desipramine intoxication	on	Desipramine	30		26
26	W	M	MDD-S	suicide, GSW to mouth	on	Fluoxetine	33		12

Race, sex, diagnosis, cause of death, medication status, antidepressant, age, pH and PMI (hours) listed for all subjects. Blank cells indicate that the information was not provided by the brain bank. Abbreviations: *W*, white; *B*, black; *F*, female; *M*, male; *CTL*, control; *MDD-NS*, major depressive disorder-non-suicide; *MDD-S*, major depressive disorder-suicide; *N/A*, not applicable; *PMI*, postmortem interval.

Table S2: TaqMan Primers and Descriptions.

Assay	Gene Symbol	Description	Assay ID
Glyceraldehyde-3-phosphate dehydrogenase	GAPDH	Housekeeping gene	Hs99999905_m1
Beta-2-microglobulin	B2M	Housekeeping gene	Hs99999907_m1
Cyclophilin A	PPIA	Housekeeping gene	Hs99999904_m1
Beta actin	ACTB	Housekeeping gene	Hs99999903_m1
Adenosine deaminase	ADA	Extracellular catabolism of adenosine	Hs01110945_m1
Adenosine A1 receptor	ADORA1	High affinity G protein coupled receptor (GPCR) for adenosine	Hs00181231_m1
Adenosine A2A receptor	ADORA2A	High affinity GPCR for adenosine	Hs00169123_m1
Alkaline phosphatase	ALPL	Generates alkaline phosphatase which dephosphorylates nucleotides	Hs01029144_m1
Ectonucleotide Pyrophosphatase / Phosphodiesterase 2	ENPP2	Encodes enzyme that catalyzes the formation of lysophosphatidic acid (LPA), which modulates purinergic receptor activity	Hs00196470_m1
Ectonucleoside triphosphate diphosphohydrolase-1	ENTPD1	Extracellular catabolism of adenosine triphosphate (ATP)	Hs00969559_m1
Ectonucleoside triphosphate diphosphohydrolase-2	ENTPD2	Extracellular catabolism of ATP	Hs00154301_m1
Ectonucleoside triphosphate diphosphohydrolase-3	ENTPD3	Extracellular catabolism of ATP	Hs00928977_m1
Ecto-5’-nucleotidase	NT5E	Rate limiting step in ATP catabolism (conversion of adenosine monophosphate to adenosine)	Hs00159686_m1
Purinergic receptor P2X 4	P2RX4	Ligand gated ion channel receptor for ATP	Hs00602442_m1
Purinergic receptor P2X 7	P2RX7	Ligand gated ion channel receptor for ATP	Hs00175721_m1
Purinergic receptor P2Y 12	P2RY12	GPCR for ATP	Hs00224470_m1
Purinergic receptor P2Y 13	P2RY13	GPCR for ATP	Hs00256749_s1
Purinergic receptor P2Y 14	P2RY14	GPCR for ATP	Hs00208434_m1
Equilibrative nucleoside transporter-1	SLC29A1	Encodes ENT1 (transport of adenosine across cell membrane)	Hs01085706_m1
Pannexin-1	PANX1	Channel that allows release of ATP into synapse	Hs00209791_m1

Assay, gene symbol, physiological description of expressed genes in vivo, and IDs for all primers (*n* = 20) utilized in the study.

Table S3: RNA Expression Profiles of Purinergic Genes in Cerebral Cortex Single Cell Types.

Assay	Gene Symbol	RNA Expression – Cell Type	Expression (nTPM)
Adenosine deaminase	ADA	Pericyte > Oligodendrocyte > Leukocyte > Astrocyte	23.2 > 17.4 > 12.0 > 11.5
Adenosine A1 receptor	ADORA1	Oligodendrocyte > OPC > Neuronal Cell (Deep Layer Near Projecting Neuron)	71.2 > 66.6 > 60.5
Adenosine A2A receptor	ADORA2A	Pericyte > Vascular SM Cell > Neuronal Cell (MSN)	89.1 > 30.8 > 29.7
Alkaline phosphatase	ALPL	Endothelial Cell	163.7
Ectonucleotide Pyrophosphatase / Phosphodiesterase 2	ENPP2	Oligodendrocyte	1508.3
Ectonucleoside triphosphate diphosphohydrolase-1	ENTPD1	CNS Macrophage > Vascular SM Cell > Endothelial Cell > Astrocyte	688.6 > 356.8 > 338.2 > 147.8
Ectonucleoside triphosphate diphosphohydrolase-2	ENTPD2	Astrocyte	50.5
Ectonucleoside triphosphate diphosphohydrolase-3	ENTPD3	Neuronal Cell (Interneuron) > Neuronal Cell (MSN)	202.0 > 151.7
Ecto-5'-nucleotidase	NT5E	Fibroblast > OPC > Astrocyte	124.9 > 86.0 > 73.5
Purinergic receptor P2X 4	P2RX4	CNS Macrophage > Leukocyte	120.6 > 47.8
Purinergic receptor P2X 7	P2RX7	Committed OPC > Oligodendrocyte > OPC > CNS Macrophage	673.9 > 383.3 > 218.4 > 215.5
Purinergic receptor P2Y 12	P2RY12	CNS Macrophage	2026.2
Purinergic receptor P2Y 13	P2RY13	CNS Macrophage	380.0
Purinergic receptor P2Y 14	P2RY14	Pericyte	1042.2
Equilibrative nucleoside transporter-1	SLC29A1	Neuronal Cell (Interneuron) > Neuronal Cell (Inhibitory Deep Layer Neuron) > Pericyte > CNS Macrophage	39.4 > 37.2 > 36.8 > 31.2
Pannexin-1	PANX1	CNS Macrophage > Leukocyte	25.3 > 19.1

Known RNA expression profiles of purinergic genes in the present study in the cerebral cortex single cell types, based on single cell and deconvolution of bulk transcriptomics. Top 1-4 cell types are displayed for each transcript along with their quantified expression value. Data extracted from the Single Cell Resource in the Human Protein Atlas (5). Abbreviations: *OPC*, oligodendrocyte precursor cell; *SM*, smooth muscle; MSN, medium spiny neuron; *nTPM*, normalized transcripts per million.

Table S4: Comparisons of Interest.

Comparisons of interest (for each cell layer, *n* = 3)	
**All Subjects**	
1	MDD-S vs MDD-NS vs CTL
**Females**	
1	MDD-S vs MDD-NS vs CTL
**Males**	
3	MDD-S vs MDD-NS vs CTL
**Medication**	
4	MDD-S – on meds vs off meds (if significant, on meds vs CTL and off meds vs CTL)
5	MDD-all subjects – on meds vs off meds (if significant, on meds vs CTL and off meds vs CTL)

Variables include diagnosis (*n* = 3), sex (*n* = 2), medication status (*n* = 2), cell layer (*n* = 3), and primer / gene of interest (*n* = 16). Abbreviations: *CTL*, control; *MDD*, major depressive disorder; *MDD-NS*, MDD-non-suicide; *MDD-S*, MDD-suicide.

Table S5: Data & Statistics—All Subject (Female & Male Combined) Comparisons (Associated with Figures 1-3).

ALL SUBJECTS						
**Number of values**	**Mean ± SEM**	**From ANCOVA or one-way ANOVA**	**P Values from Post-Hoc Test**	**From two-way ANOVA—Main effect of Sex**	**From two-way ANOVA—Main effect of Disease**	**P Values from Tukey's Post-Hoc Test**
**ENTPD2 (Superficial Gray Matter)**						
9 (CTL)<br>6 (NS)<br>10 (S)	-0.09 ± 0.10 (CTL)<br>0.22 ± 0.98 (NS)<br>-0.05 ± 0.09 (S)	F (2, 21) =<br>0.37, P = 0.69<br>(age-adjusted)	0.10 (CTL v NS)<br>0.95 (CTL v S)<br>0.14 (NS v S)	F (9, 13) = 0.68,<br>P = 0.72	F (2, 13) = 2.10,<br>P = 0.16	0.21 (CTL v NS)<br>0.99 (CTL v S)<br>0.18 (NS v S)
**ENTPD2 (Deep Gray Matter)**						
9 (CTL)<br>6 (NS)<br>10 (S)	-0.08 ± 0.10 (CTL)<br>0.24 ± 0.06 (NS)<br>-0.08 ± 0.12 (S)	F (2, 21) =<br>0.39, P = 0.68<br>(age-adjusted)	0.23 (CTL v NS)<br>>0.99 (CTL v S)<br>0.14 (NS v S)	F (9, 13) = 1.27,<br>P = 0.34	F (2, 13) = 3.61,<br>P = 0.06	0.14 (CTL v NS)<br>0.77 (CTL v S)<br>*** 0.05 (NS v S)**
**ENTPD2 (White Matter)**						
10 (CTL)<br>6 (NS)<br>10 (S)	-0.07 ± 0.18 (CTL)<br>0.07 ± 0.19 (NS)<br>-0.12 ± 0.11 (S)	F (2, 23) =<br>0.15,<br>P = 0.86	0.85 (CTL v NS)<br>0.97 (CTL v S)<br>0.94 (NS v S)	F (9, 14) = 0.39,<br>P = 0.92	F (2, 14) = 0.09,<br>P = 0.91	0.91 (CTL v NS)<br>0.98 (CTL v S)<br>0.96 (NS v S)
**ENTPD3 (Superficial Gray Matter)**						
9 (CTL)<br>6 (NS)<br>10 (S)	-0.11 ± 0.03 (CTL)<br>-0.17 ± 0.14 (NS)<br>-0.01 ± 0.03 (S)	F (2, 21) =<br>2.51, P = 0.11<br>(age-adjusted)	0.96 (CTL v NS)<br>0.12 (CTL v S)<br>0.66 (NS v S)	F (9, 13) = 0.87,<br>P = 0.58	F (2, 13) = 0.99,<br>P = 0.39	0.84 (CTL v NS)<br>0.63 (CTL v S)<br>0.39 (NS v S)
**ENTPD3 (Deep Gray Matter)**						
9 (CTL)<br>6 (NS)<br>10 (S)	-0.08 ± 0.05 (CTL)<br>-0.11 ± 0.12 (NS)<br>0.08 ± 0.07 (S)	F (2,22) =<br>2.08,<br>P = 0.15	0.96 (CTL v NS)<br>0.23 (CTL v S)<br>0.21 (NS v S)	F (9, 13) = 2.61,<br>P = 0.06	F (2, 13) = 1.52,<br>P = 0.26	0.95 (CTL v NS)<br>0.35 (CTL v S)<br>0.32 (NS v S)
**ENTPD3 (White Matter)**						
10 (CTL)<br>6 (NS)<br>10 (S)	-0.21 ± 0.13 (CTL)<br>-0.04 ± 0.20 (NS)<br>0.14 ± 0.20 (S)	F (2, 22) =<br>0.98, P = 0.39<br>(PMI-adjusted)	0.92 (CTL v NS)<br>0.11 (CTL v S)<br>>0.99 (NS v S)	F (9, 14) = 1.80,<br>P = 0.16	F (2, 14) = 1.44,<br>P = 0.27	0.90 (CTL v NS)<br>0.25 (CTL v S)<br>0.63 (NS v S)
**NT5E (Superficial Gray Matter)**						
9 (CTL)<br>6 (NS)<br>10 (S)	-0.21 ± 0.06 (CTL)<br>-0.13 ± 0.04 (NS)<br>-0.16 ± 0.06 (S)	F (2, 22) =<br>0.54,<br>P = 0.59	0.59 (CTL v NS)<br>0.75 (CTL v S)<br>0.93 (NS v S)	F (9, 13) = 0.52,<br>P = 0.83	F (2, 13) = 048,<br>P = 0.63	0.62 (CTL v NS)<br>0.82 (CTL v S)<br>0.91 (NS v S)
**NT5E (Deep Gray Matter)**						
9 (CTL)<br>6 (NS)<br>10 (S)	0.02 ± 0.07 (CTL)<br>0.07 ± 0.06 (NS)<br>0.01 ± 0.05 (S)	F (2, 22) =<br>0.25,<br>P = 0.78	0.84 (CTL v NS)<br>0.99 (CTL v S)<br>0.77 (NS v S)	F (9, 13) = 0.44,<br>P = 0.88	F (2, 13) = 0.35,<br>P = 0.71	0.83 (CTL v NS)<br>0.96 (CTL v S)<br>0.69 (NS v S)
**NT5E (White Matter)**						
10 (CTL)<br>6 (NS)<br>10 (S)	-0.07 ± 0.11 (CTL)<br>-0.13 ± 0.07 (NS)<br>0.01 ± 0.12 (S)	F (2, 23) =<br>0.36,<br>P = 0.70	0.95 (CTL v NS)<br>0.83 (CTL v S)<br>0.70 (NS v S)	F (9, 14) = 0.27,<br>P = 0.97	F (2, 14) = 0.24,<br>P = 0.79	0.97 (CTL v NS)<br>0.87 (CTL v S)<br>0.80 (NS v S)
**PANX1 (Superficial Gray Matter)**						
9 (CTL)<br>6 (NS)<br>10 (S)	-0.05 ± 0.02 (CTL)<br>-0.09 ± 0.10 (NS)<br>-0.02 ± 0.02 (S)	W = 0.78,<br>P = 0.48	0.97 (CTL v NS)<br>0.59 (CTL v S)<br>0.84 (NS v S)	F (9, 13) = 0.94,<br>P = 0.52	F (2, 13) = 0.35,<br>P = 0.71	0.94 (CTL v NS)<br>0.84 (CTL v S)<br>0.71 (NS v S)
**PANX1 (Deep Gray Matter)**						
9 (CTL)<br>6 (NS)<br>10 (S)	0.06 ± 0.06 (CTL)<br>-0.07 ± 0.14 (NS)<br>0.05 ± 0.05 (S)	KW = 1.65,<br>P = 0.44	0.61 (CTL v NS)<br>>0.99 (CTL v S)<br>>0.99 (NS v S)	F (9, 13) = 1.06,<br>P = 0.45	F (2, 13) = 0.46,<br>P = 0.64	0.61 (CTL v NS)<br>0.92 (CTL v S)<br>0.81 (NS v S)
**PANX1 (White Matter)**						
10 (CTL)<br>6 (NS)<br>10 (S)	-0.06 ± 0.07 (CTL)<br>-0.03 ± 0.10 (NS)<br>0.23 ± 0.09 (S)	F (2, 22) =<br>5.86, *P = 0.01<br>(PMI-adjusted)	>0.99 (CTL v NS)<br>*** 0.02 (CTL v S)**<br>0.08 (NS v S)	F (9, 14) = 1.69,<br>P = 0.18	F (2, 14) = 4.96,<br>*** P = 0.02**	0.98 (CTL v NS)<br>*** 0.03 (CTL v S)**<br>0.10 (NS v S)
**SLC29A1 (Superficial Gray Matter)**						
9 (CTL)<br>6 (NS)<br>10 (S)	0.03 ± 0.06 (CTL)<br>0.04 ± 0.07 (NS)<br>0.20 ± 0.07 (S)	F (2, 22) =<br>2.04,<br>P = 0.15	0.99 (CTL v NS)<br>0.18 (CTL v S)<br>0.29 (NS v S)	F (9, 13) = 4.77,<br>*** P = 0.01**	F (2, 13) = 1.86,<br>P = 0.20	0.72 (CTL v NS)<br>0.17 (CTL v S)<br>0.68 (NS v S)
**SLC29A1 (Deep Gray Matter)**						
9 (CTL)<br>6 (NS)<br>10 (S)	0.02 ± 0.06 (CTL)<br>-0.05 ± 0.09 (NS)<br>0.23 ± 0.14 (S)	W = 1.33,<br>P = 0.30	0.86 (CTL v NS)<br>0.49 (CTL v S)<br>0.30 (NS v S)	F (9, 13) = 2.97,<br>*** P = 0.04**	F (2, 13) = 0.55,<br>P = 0.59	0.96 (CTL v NS)<br>0.71 (CTL v S)<br>0.62 (NS v S)
**SLC29A1 (White Matter)**						
10 (CTL)<br>6 (NS)<br>10 (S)	0.12 ± 0.08 (CTL)<br>0.12 ± 0.09 (NS)<br>0.43 ± 0.14 (S)	F (2, 23) =<br>2.73,<br>P = 0.09	0.99 (CTL v NS)<br>0.11 (CTL v S)<br>0.18 (NS v S)	F (9, 14) = 1.30,<br>P = 0.32	F (2, 14) = 2.91,<br>P = 0.09	>0.99 (CTL v NS)<br>0.10 (CTL v S)<br>0.21 (NS v S)
**ADA (Superficial Gray Matter)**						
9 (CTL)<br>6 (NS)<br>10 (S)	-0.01 ± 0.06 (CTL)<br>0.27 ± 0.13 (NS)<br>0.06 ± 0.09 (S)	KW = 3.671,<br>P = 0.16	0.18 (CTL v NS)<br>>0.99 (CTL v S)<br>0.44 (NS v S)	F (9, 13) = 0.99,<br>P = 0.49	F (2, 13) = 1.62,<br>P = 0.23	0.24 (CTL v NS)<br>0.98 (CTL v S)<br>0.30 (NS v S)
**ADA (Deep Gray Matter)**						

9 (CTL)<br>6 (NS)<br>10 (S)	0.08 ± 0.08 (CTL)<br>0.11 ± 0.12 (NS)<br>0.22 ± 0.17 (S)	F (2, 22) =<br>0.27, P = 0.77<br>(PMI-adjusted	>0.99 (CTL v NS)<br>>0.99 (CTL v S)<br>>0.99 (NS v S)	F (9, 13) = 2.68, P<br>= 0.05	F (2, 13) = 0.05, P<br>= 0.95	0.95 (CTL v NS)<br>0.99 (CTL v S)<br>0.97 (NS v S)
**ADA (White Matter)**						
10 (CTL)<br>6 (NS)<br>10 (S)	0.04 ± 0.13 (CTL)<br>-0.20 ± 0.30 (NS)<br>0.25 ± 0.09 (S)	KW = 6.42,<br>* P = 0.04	0.65 (CTL v NS)<br>0.43 (CTL v S)<br>*** 0.04 (NS v S)**	F (9, 14) = 0.88,<br>P = 0.57	F (2, 14) = 3.80,<br>*** P = 0.05**	0.28 (CTL v NS)<br>0.37 (CTL v S)<br>*** 0.04 (NS v S)**
**P2RX4 (Superficial Gray Matter)**						
9 (CTL)<br>6 (NS)<br>10 (S)	-0.09 ± 0.07 (CTL)<br>0.05 ± 0.08 (NS)<br>0.01 ± 0.09 (S)	F (2,22) =<br>0.77,<br>P = 0.48	0.49 (CTL v NS)<br>0.62 (CTL v S)<br>0.94 (NS v S)	F (9, 13) = 2.43,<br>P = 0.07	F (2, 13) = 1.43,<br>P = 0.27	0.26 (CTL v NS)<br>0.92 (CTL v S)<br>0.41 (NS v S)
**P2RX4 (Deep Gray Matter)**						
9 (CTL)<br>6 (NS)<br>10 (S)	0.14 ± 0.09 (CTL)<br>0.08 ± 0.06 (NS)<br>0.27 ± 0.13 (S)	F (2, 22) =<br>0.71,<br>P = 0.50	0.93 (CTL v NS)<br>0.68 (CTL v S)<br>0.51 (NS v S)	F (9, 13) = 2.50,<br>P = 0.07	F (2, 13) = 0.09,<br>P = 0.92	0.97 (CTL v NS)<br>0.98 (CTL v S)<br>0.91 (NS v S)
**P2RX4 (White Matter)**						
10 (CTL)<br>6 (NS)<br>10 (S)	0.20 ± 0.07 (CTL)<br>-0.04 ± 0.11 (NS)<br>0.36 ± 0.08 (S)	KW = 8.81,<br>* P = 0.01	0.36 (CTL v NS)<br>0.32 (CTL v S)<br>*** 0.01 (NS v S)**	F (9, 14) = 0.48,<br>P = 0.87	F (2, 14) = 4.46,<br>*** P = 0.03**	0.20 (CTL v NS)<br>0.36 (CTL v S)<br>*** 0.03 (NS v S)**
**P2RY12 (Superficial Gray Matter)**						
9 (CTL)<br>6 (NS)<br>10 (S)	-0.15 ± 0.08 (CTL)<br>-0.45 ± 0.22 (NS)<br>-0.18 ± 0.11 (S)	F (2, 21) =<br>0.07, P = 0.64<br>(age-adjusted)	>0.99 (CTL v NS)<br>>0.99 (CTL v S)<br>>0.99 (NS v S)	F (9, 13) = 0.66,<br>P = 0.73	F (2, 13) = 1.09,<br>P = 0.37	0.36 (CTL v NS)<br>0.98 (CTL v S)<br>0.46 (NS v S)
**P2RY12 (Deep Gray Matter)**						
9 (CTL)<br>6 (NS)<br>10 (S)	-0.08 ± 0.09 (CTL)<br>-0.60 ± 0.22 (NS)<br>-0.21 ± 0.10 (S)	F (2, 22) =<br>3.69,<br>* P = 0.04	*** 0.04 (CTL v NS)**<br>0.74 (CTL v S)<br>0.12 (NS v S)	F (9, 13) = 1.23,<br>P = 0.36	F (2, 13) = 3.52,<br>P = 0.06	*** 0.05 (CTL v NS)**<br>0.53 (CTL v S)<br>0.24 (NS v S)
**P2RY12 (White Matter)**						
10 (CTL)<br>6 (NS)<br>10 (S)	-0.19 ± 0.09 (CTL)<br>-0.83 ± 0.31 (NS)<br>-0.08 ± 0.12 (S)	F (2, 22) =<br>1.89, P = 0.17<br>(age-adjusted)	0.24 (CTL v NS)<br>0.83 (CTL v S)<br>0.17 (NS v S)	F (9, 14) = 1.22,<br>P = 0.34	F (2, 14) = 5.82,<br>*** P = 0.01**	*** 0.03 (CTL v NS)**<br>0.84 (CTL v S)<br>*** 0.01 (NS v S)**
**P2RY13 (Superficial Gray Matter)**						
9 (CTL)<br>6 (NS)<br>10 (S)	-0.11 ± 0.04 (CTL)<br>-0.07 ± 0.13 (NS)<br>0.07 ± 0.06 (S)	KW = 5.17,<br>P = 0.08	>0.99 (CTL v NS)<br>0.13 (CTL v S)<br>0.23 (NS v S)	F (9, 13) = 0.01,<br>P = 0.49	F (2, 13) = 1.03,<br>P = 0.39	0.89 (CTL v NS)<br>0.36 (CTL v S)<br>0.73 (NS v S)
**P2RY13 (Deep Gray Matter)**						
9 (CTL)<br>6 (NS)<br>10 (S)	-0.04 ± 0.07 (CTL)<br>-0.06 ± 0.09 (NS)<br>0.09 ± 0.09 (S)	KW = 2.59,<br>P = 0.27	>0.99 (CTL v NS)<br>0.87 (CTL v S)<br>0.37 (NS v S)	F (9, 13) = 1.43,<br>P = 0.27	F (2, 13) = 0.30,<br>P = 0.75	0.99 (CTL v NS)<br>0.81 (CTL v S)<br>0.78 (NS v S)
**P2RY13 (White Matter)**						
10 (CTL)<br>6 (NS)<br>10 (S)	0.04 ± 0.07 (CTL)<br>-0.25 ± 0.09 (NS)<br>0.20 ± 0.06 (S)	F (2, 22) =<br>4.62, *P = 0.02<br>(age-adjusted)	*** 0.04 (CTL v NS)**<br>0.23 (CTL v S)<br>*** 0.00 (NS v S)**	F (9, 14) = 0.28,<br>P = 0.97	F (2, 14) = 6.54,<br>*** P = 0.01**	0.08 (CTL v NS)<br>0.29 (CTL v S)<br>*** 0.01 (NS v S)**

Number of values, means, and standard error of the mean (SEM) values for control (CTL), major depressive disorder—non-suicide (MDD-NS), and major depressive disorder—suicide (MDD-S) groups reported individually for gene transcripts and cell layers. Results from analysis of covariance (ANCOVA) are reported when a significant covariate effect (age or postmortem interval, PMI) was detected; otherwise, one-way analysis of variance (ANOVA) results are presented. Two-way ANOVA results are also included. P-values for all primary analyses and post hoc tests, corrected for multiple comparisons, are reported: Bonferroni for ANCOVA, Tukey's for one-way and two-way ANOVA, Dunn's for the Kruskal-Wallis nonparametric test, and Dunnett's T3 for Welch's-corrected ANOVA. Note for the two-way ANOVA: an interaction term could not be calculated due to the lack of replicates; thus, the results only reflect the independent main effects of sex and disease. Red text indicates statistically significant findings (* $p < 0.05$). Data shown for all subjects (females and males combined) comparison group. Abbreviations: *KW*, Kruskal-Wallis statistic; *W*, Welch's corrected statistic; Assays associated with gene symbols are reported in Table S2.

Table S6: Data & Statistics—Female Only & Male Only Comparisons (Associated with Figures 1-3).

FEMALE SUBJECTS				MALE SUBJECTS			
**Number of values**	**Mean ± SEM**	**From ANCOVA or one-way ANOVA**	**P Values from Post-Hoc Test**	**Number of values**	**Mean ± SEM**	**From ANCOVA or one-way ANOVA**	**P Values from Post-Hoc Test**
**ENTPD2 (Superficial Gray Matter)**							
5 (CTL)<br>3 (NS)<br>5 (S)	-0.31 ± 0.09 (CTL)<br>0.26 ± 0.11 (NS)<br>+0.02 ± 0.09 (S)	F (2, 10) = 7.30,<br>* P = 0.01	*** 0.01 (CTL v NS)**<br>0.11 (CTL v S)<br>0.21 (NS v S)	4 (CTL)<br>3 (NS)<br>5 (S)	0.18 ± 0.06 (CTL)<br>0.18 ± 0.13 (NS)<br>-0.09 ± 0.16 (S)	F (2, 8) = 0.75, P = 0.50 (age-adjusted)	0.99 (CTL v NS)<br>0.34 (CTL v S)<br>0.38 (NS v S)
**ENTPD2 (Deep Gray Matter)**							
5 (CTL)<br>3 (NS)<br>5 (S)	-0.32 ± 0.04 (CTL)<br>0.22 ± 0.12 (NS)<br>-0.02 ± 0.12 (S)	F (2, 10) = 7.37,<br>P = * 0.01	*** 0.01 (CTL v NS)**<br>0.09 (CTL v S)<br>0.25 (NS v S)	4 (CTL)<br>3 (NS)<br>5 (S)	0.22 ± 0.05 (CTL)<br>0.27 ± 0.06 (NS)<br>-0.14 ± 0.23 (S)	F (2, 9) = 1.78,<br>P = 0.22	0.98 (CTL v NS)<br>0.32 (CTL v S)<br>0.29 (NS v S)
**ENTPD2 (White Matter)**							
5 (CTL)<br>3 (NS)<br>5 (S)	-0.43 ± 0.18 (CTL)<br>0.07 ± 0.27 (NS)<br>0.26 ± 0.06 (S)	F (2, 10) = 5.20,<br>* P = 0.03	0.17 (CTL v NS)<br>*** 0.03 (CTL v S)**<br>0.73 (NS v S)	5 (CTL)<br>3 (NS)<br>5 (S)	0.30 ± 0.22 (CTL)<br>0.07 ± 0.31 (NS)<br>-0.30 ± 0.12 (S)	F (2, 10) = 2.43,<br>P = 0.14	0.75 (CTL v NS)<br>0.12 (CTL v S)<br>0.50 (NS v S)
**ENTPD3 (Superficial Gray Matter)**							
5 (CTL)<br>3 (NS)<br>5 (S)	-0.09 ± 0.03 (CTL)<br>-0.23 ± 0.17 (NS)<br>-0.07 ± 0.02 (S)	F (2, 10) = 1.36,<br>P = 0.30	0.38 (CTL v NS)<br>0.98 (CTL v S)<br>0.31 (NS v S)	4 (CTL)<br>3 (NS)<br>5 (S)	-0.13 ± 0.06 (CTL)<br>-0.10 ± 0.26 (NS)<br>0.04 ± 0.04 (S)	F (2, 9) = 0.69,<br>P = 0.52	0.99 (CTL v NS)<br>0.55 (CTL v S)<br>0.68 (NS v S)
**ENTPD3 (Deep Gray Matter)**							
5 (CTL)<br>3 (NS)<br>5 (S)	-0.13 ± 0.05 (CTL)<br>-0.19 ± 0.20 (NS)<br>-0.07 ± 0.04 (S)	F (2, 10) = 0.45,<br>P = 0.65	0.88 (CTL v NS)<br>0.86 (CTL v S)<br>0.63 (NS v S)	4 (CTL)<br>3 (NS)<br>5 (S)	-0.03 ± 0.08 (CTL)<br>-0.03 ± 0.15 (NS)<br>0.23 ± 0.09 (S)	F (2, 9) = 2.27,<br>P = 0.16	>0.99 (CTL v NS)<br>0.21 (CTL v S)<br>0.25 (NS v S)
**ENTPD3 (White Matter)**							
5 (CTL)<br>3 (NS)<br>5 (S)	-0.37 ± 0.08 (CTL)<br>0.22 ± 0.10 (NS)<br>0.48 ± 0.09 (S)	F (2, 10) = 28.99,<br>* P = 0.00	*** 0.00 (CTL v NS)**<br>*** 0.00 (CTL v S)**<br>0.17 (NS v S)	5 (CTL)<br>3 (NS)<br>5 (S)	-0.04 ± 0.24 (CTL)<br>-0.31 ± 0.34 (NS)<br>-0.21 ± 0.34 (S)	F (2, 10) = 0.18,<br>P = 0.84	0.84 (CTL v NS)<br>0.91 (CTL v S)<br>0.97 (NS v S)
**NT5E (Superficial Gray Matter)**							
5 (CTL)<br>3 (NS)<br>5 (S)	-0.32 ± 0.05 (CTL)<br>-0.09 ± 0.05 (NS)<br>-0.07 ± 0.09 (S)	F (2, 10) = 3.61,<br>P = 0.07	0.18 (CTL v NS)<br>0.07 (CTL v S)<br>0.96 (NS v S)	4 (CTL)<br>3 (NS)<br>5 (S)	-0.08 ± 0.09 (CTL)<br>-0.15 ± 0.07 (NS)<br>-0.25 ± 0.06 (S)	F (2, 9) = 1.44,<br>P = 0.29	0.82 (CTL v NS)<br>0.26 (CTL v S)<br>0.65 (NS v S)
**NT5E (Deep Gray Matter)**							
5 (CTL)<br>3 (NS)<br>5 (S)	-0.11 ± 0.06 (CTL)<br>0.10 ± 0.02 (NS)<br>0.10 ± 0.05 (S)	F (2, 9) = 3.77, * P = 0.06 (age-adjusted)	0.07 (CTL v NS)<br>0.06 (CTL v S)<br>>0.99 (NS v S)	4 (CTL)<br>3 (NS)<br>5 (S)	0.19 ± 0.10 (CTL)<br>0.05 ± 0.15 (NS)<br>-0.08 ± 0.07 (S)	F (2, 9) = 2.19,<br>P = 0.17	0.65 (CTL v NS)<br>0.15 (CTL v S)<br>0.62 (NS v S)
**NT5E (White Matter)**							
5 (CTL)<br>3 (NS)<br>5 (S)	-0.34 ± 0.10 (CTL)<br>-0.11 ± 0.15 (NS)<br>0.32 ± 0.06 (S)	F (2, 10) = 13.37,<br>* P = 0.00	0.31 (CTL v NS)<br>*** 0.00 (CTL v S)**<br>*** 0.04 (NS v S)**	5 (CTL)<br>3 (NS)<br>5 (S)	0.19 ± 0.07 (CTL)<br>-0.14 ± 0.05 (NS)<br>-0.30 ± 0.11 (S)	F (2, 10) = 8.90,<br>* P = 0.01	0.08 (CTL v NS)<br>*** 0.01 (CTL v S)**<br>0.50 (NS v S)
**PANX1 (Superficial Gray Matter)**							
5 (CTL)<br>3 (NS)<br>5 (S)	-0.03 ± 0.03 (CTL)<br>-0.24 ± 0.10 (NS)<br>-0.04 ± 0.03 (S)	F (2, 10) = 4.66,<br>* P = 0.04	*** 0.05 (CTL v NS)**<br>0.99 (CTL v S)<br>*** 0.05 (NS v S)**	4 (CTL)<br>3 (NS)<br>5 (S)	-0.08 ± 0.04 (CTL)<br>0.06 ± 0.13 (NS)<br>0.01 ± 0.03 (S)	F (2, 9) = 0.99,<br>P = 0.41	0.40 (CTL v NS)<br>0.62 (CTL v S)<br>0.85 (NS v S)
**PANX1 (Deep Gray Matter)**							
5 (CTL)<br>3 (NS)<br>5 (S)	0.03 ± 0.10 (CTL)<br>-0.25 ± 0.21 (NS)<br>-0.02 ± 0.07 (S)	KW = 2.85,<br>P = 0.26	0.27 (CTL v NS)<br>>0.99 (CTL v S)<br>0.87 (NS v S)	4 (CTL)<br>3 (NS)<br>5 (S)	0.11 ± 0.05 (CTL)<br>0.11 ± 0.13 (NS)<br>0.12 ± 0.05 (S)	F (2, 8) = 0.58, P = 0.58 (age-adjusted)	>0.99 (CTL v NS)<br>0.99 (CTL v S)<br>>0.99 (NS v S)
**PANX1 (White Matter)**							
5 (CTL)<br>3 (NS)<br>5 (S)	-0.11 ± 0.07 (CTL)<br>0.14 ± 0.06 (NS)<br>0.42 ± 0.08 (S)	F (2, 9) = 13.96, *P = 0.00 (PMI-adjusted)	0.05 (CTL v NS)<br>*** 0.00 (CTL v S)**<br>*** 0.03 (NS v S)**	5 (CTL)<br>3 (NS)<br>5 (S)	-0.02 ± 0.12 (CTL)<br>-0.21 ± 0.14 (NS)<br>0.04 ± 0.10 (S)	F (2, 10) = 0.98,<br>P = 0.41	0.55 (CTL v NS)<br>0.93 (CTL v S)<br>0.39 (NS v S)
**SLC29A1 (Superficial Gray Matter)**							
5 (CTL)<br>3 (NS)<br>5 (S)	0.08 ± 0.09 (CTL)<br>0.03 ± 0.07 (NS)<br>0.16 ± 0.09 (S)	F (2, 10) = 0.52,<br>P = 0.61	0.93 (CTL v NS)<br>0.76 (CTL v S)<br>0.61 (NS v S)	4 (CTL)<br>3 (NS)<br>5 (S)	-0.02 ± 0.08 (CTL)<br>0.05 ± 0.14 (NS)<br>0.24 ± 0.12 (S)	KW = 3.31,<br>P = 0.20	>0.99 (CTL v NS)<br>0.22 (CTL v S)<br>0.97 (NS v S)
**SLC29A1 (Deep Gray Matter)**							
5 (CTL)<br>3 (NS)<br>5 (S)	-0.04 ± 0.08 (CTL)<br>-0.03 ± 0.06 (NS)<br>0.14 ± 0.10 (S)	F (2, 10) = 1.40,<br>P = 0.29	>0.99 (CTL v NS)<br>0.32 (CTL v S)<br>0.44 (NS v S)	4 (CTL)<br>3 (NS)<br>5 (S)	0.10 ± 0.10 (CTL)<br>-0.08 ± 0.20 (NS)<br>0.31 ± 0.27 (S)	F (2, 9) = 0.73,<br>P = 0.51	0.87 (CTL v NS)<br>0.77 (CTL v S)<br>0.49 (NS v S)
**SLC29A1 (White Matter)**							
5 (CTL)<br>3 (NS)<br>5 (S)	0.03 ± 0.11 (CTL)<br>0.19 ± 0.01 (NS)<br>0.59 ± 0.11 (S)	F (2, 9) = 7.15, *P = 0.01 (PMI-adjusted)	0.94 (CTL v NS)<br>*** 0.01 (CTL v S)**<br>0.07 (NS v S)	5 (CTL)<br>3 (NS)<br>5 (S)	0.21 ± 0.10 (CTL)<br>0.04 ± 0.18 (NS)<br>0.26 ± 0.24 (S)	F (2, 10) = 0.29,<br>P = 0.75	0.83 (CTL v NS)<br>0.98 (CTL v S)<br>0.74 (NS v S)
**ADA (Superficial Gray Matter)**							

5 (CTL)<br>3 (NS)<br>5 (S)	-0.06 ± 0.08 (CTL)<br>0.32 ± 0.27 (NS)<br>-0.01 ± 0.12 (S)	KW = 2.88,<br>P = 0.25	0.33 (CTL v NS)<br>>0.99 (CTL v S)<br>0.44 (NS v S)	4 (CTL)<br>3 (NS)<br>5 (S)	0.05 ± 0.10 (CTL)<br>0.22 ± (NS)<br>0.13 ± 0.14 (S)	F (2, 8) =<br>2.32, P =<br>0.16 (PMI-<br>adjusted)	0.66 (CTL v NS)<br>0.89 (CTL v S)<br>0.87 (NS v S)
**ADA (Deep Gray Matter)**							
5 (CTL)<br>3 (NS)<br>5 (S)	0.08 ± 0.11 (CTL)<br>0.09 ± 0.23 (NS)<br>-0.01 ± 0.18 (S)	KW = 1.93,<br>P = 0.41	>0.99 (CTL v NS)<br>0.50 (CTL v S)<br>>0.99 (NS v S)	4 (CTL)<br>3 (NS)<br>5 (S)	0.09 ± 0.15 (CTL)<br>0.15 ± 0.14 (NS)<br>0.44 ± 0.28 (S)	F (2, 9) =<br>0.75,<br>P = 0.50	0.99 (CTL v NS)<br>0.52 (CTL v S)<br>0.67 (NS v S)
**ADA (White Matter)**							
5 (CTL)<br>3 (NS)<br>5 (S)	0.12 ± 0.25 (CTL)<br>-0.18 ± 0.13 (NS)<br>0.12 ± 0.12 (S)	F (2, 10) =<br>0.63,<br>P = 0.55	0.59 (CTL v NS)<br>>0.99 (CTL v S)<br>0.58 (NS v S)	5 (CTL)<br>3 (NS)<br>5 (S)	-0.04 ± 0.08 (CTL)<br>-0.22 ± 0.24 (NS)<br>0.38 ± 0.12 (S)	F (2, 9) =<br>2.84, P =<br>0.11 (PMI-<br>adjusted)	>0.99 (CTL v NS)<br>0.19 (CTL v S)<br>0.09 (NS v S)
**P2RX4 (Superficial Gray Matter)**							
5 (CTL)<br>3 (NS)<br>5 (S)	-0.03 ± 0.07 (CTL)<br>0.11 ± 0.15 (NS)<br>-0.13 ± 0.09 (S)	F (2, 10) =<br>1.31,<br>P = 0.31	0.65 (CTL v NS)<br>0.69 (CTL v S)<br>0.28 (NS v S)	4 (CTL)<br>3 (NS)<br>5 (S)	-0.19 ± 0.11 (CTL)<br>-0.01 ± 0.07 (NS)<br>0.14 ± 0.15 (S)	KW = 1.41,<br>P = 0.54	>0.99 (CTL v NS)<br>0.82 (CTL v S)<br>>0.99 (NS v S)
**P2RX4 (Deep Gray Matter)**							
5 (CTL)<br>3 (NS)<br>5 (S)	0.04 ± 0.14 (CTL)<br>0.14 ± 0.11 (NS)<br>0.08 ± 0.09 (S)	F (2, 10) =<br>0.15,<br>P = 0.86	0.85 (CTL v NS)<br>0.97 (CTL v S)<br>0.93 (NS v S)	4 (CTL)<br>3 (NS)<br>5 (S)	0.27 ± 0.10 (CTL)<br>0.02 ± 0.06 (NS)<br>0.46 ± 0.23 (S)	F (2, 8) =<br>0.22, P =<br>0.81 (PMI-<br>adjusted)	0.66 (CTL v NS)<br>0.71 (CTL v S)<br>0.27 (NS v S)
**P2RX4 (White Matter)**							
5 (CTL)<br>3 (NS)<br>5 (S)	0.19 ± 0.13 (CTL)<br>0.05 ± 0.03 (NS)<br>0.31 ± 0.06 (S)	KW = 5.50,<br>P = 0.06	>0.99 (CTL v NS)<br>0.31 (CTL v S)<br>0.08 (NS v S)	5 (CTL)<br>3 (NS)<br>5 (S)	0.21 ± 0.04 (CTL)<br>-0.13 ± 0.22 (NS)<br>0.42 ± 0.15 (S)	F (2, 9) =<br>1.07, P =<br>0.38 (PMI-<br>adjusted)	0.24 (CTL v NS)<br>0.47 (CTL v S)<br>0.38 (NS v S)
**P2RY12 (Superficial Gray Matter)**							
5 (CTL)<br>3 (NS)<br>5 (S)	-0.14 ± 0.06 (CTL)<br>-0.37 ± 0.40 (NS)<br>-0.19 ± 0.21 (S)	KW = 0.66,<br>P = 0.75	>0.99 (CTL v NS)<br>>0.99 (CTL v S)<br>>0.99 (NS v S)	4 (CTL)<br>3 (NS)<br>5 (S)	-0.17 ± 0.18 (CTL)<br>-0.53 ± 0.29 (NS)<br>-0.16 ± 0.12 (S)	F (2, 9) =<br>1.13,<br>P = 0.37	0.43 (CTL v NS)<br>>0.99 (CTL v S)<br>0.39 (NS v S)
**P2RY12 (Deep Gray Matter)**							
5 (CTL)<br>3 (NS)<br>5 (S)	-0.15 ± 0.10 (CTL)<br>-0.51 ± 0.40 (NS)<br>-0.26 ± 0.17 (S)	F (2, 10) =<br>0.71,<br>P = 0.51	0.49 (CTL v NS)<br>0.90 (CTL v S)<br>0.70 (NS v S)	4 (CTL)<br>3 (NS)<br>5 (S)	0.00 ± 0.17 (CTL)<br>-0.68 ± 0.26 (NS)<br>-0.15 ± 0.13 (S)	F (2, 9) =<br>3.56,<br>P = 0.07	0.07 (CTL v NS)<br>0.80 (CTL v S)<br>0.15 (NS v S)
**P2RY12 (White Matter)**							
5 (CTL)<br>3 (NS)<br>5 (S)	-0.22 ± 0.14 (CTL)<br>-0.85 ± 0.32 (NS)<br>0.01 ± 0.22 (S)	F (2, 10) =<br>3.50,<br>P = 0.07	0.18 (CTL v NS)<br>0.72 (CTL v S)<br>0.06 (NS v S)	5 (CTL)<br>3 (NS)<br>5 (S)	-0.16 ± 0.12 (CTL)<br>-0.81 ± 0.63 (NS)<br>-0.17 ± 0.08 (S)	F (2, 10) =<br>1.74,<br>P = 0.26	0.26 (CTL v NS)<br>>0.99 (CTL v S)<br>0.26 (NS v S)
**P2RY13 (Superficial Gray Matter)**							
5 (CTL)<br>3 (NS)<br>5 (S)	-0.20 ± 0.02 (CTL)<br>-0.22 ± 0.07 (NS)<br>0.04 ± 0.07 (S)	F (2, 10) =<br>7.11,<br>* P = 0.01	0.96 (CTL v NS)<br>*** 0.02 (CTL v S)**<br>*** 0.03 (NS v S)**	4 (CTL)<br>3 (NS)<br>5 (S)	0.00 ± 0.06 (CTL)<br>0.07 ± 0.24 (NS)<br>0.09 ± 0.10 (S)	F (2, 9) =<br>0.15,<br>P = 0.86	0.93 (CTL v NS)<br>0.86 (CTL v S)<br>0.99 (NS v S)
**P2RY13 (Deep Gray Matter)**							
5 (CTL)<br>3 (NS)<br>5 (S)	-0.04 ± 0.09 (CTL)<br>-0.18 ± 0.05 (NS)<br>-0.00 ± 0.05 (S)	F (2, 9) =<br>0.72, P =<br>0.51 (age-<br>adjusted)	0.47 (CTL v NS)<br>0.91 (CTL v S)<br>0.30 (NS v S)	4 (CTL)<br>3 (NS)<br>5 (S)	-0.05 ± 0.12 (CTL)<br>0.06 ± 0.17 (NS)<br>0.18 ± 0.17 (S)	F (2, 9) =<br>0.59,<br>P = 0.57	0.89 (CTL v NS)<br>0.54 (CTL v S)<br>0.86 (NS v S)
**P2RY13 (White Matter)**							
5 (CTL)<br>3 (NS)<br>5 (S)	-0.00 ± 0.14 (CTL)<br>-0.38 ± 0.13 (NS)<br>0.27 ± 0.09 (S)	F (2, 9) =<br>3.99, P =<br>0.06 (age-<br>adjusted)	0.15 (CTL v NS)<br>0.28 (CTL v S)<br>*** 0.01 (NS v S)**	5 (CTL)<br>3 (NS)<br>5 (S)	0.08 ± 0.03 (CTL)<br>-0.11 ± 0.07 (NS)<br>0.14 ± 0.06 (S)	F (2, 10) =<br>4.99,<br>* P = 0.03	0.10 (CTL v NS)<br>0.63 (CTL v S)<br>*** 0.03 (NS v S)**

Number of values, means, and standard error of the mean (SEM) values for control (CTL), major depressive disorder—non-suicide (MDD-NS), and major depressive disorder—suicide (MDD-S) groups reported individually for sex, gene transcripts, and cell layers. Results from analysis of covariance (ANCOVA) are reported when a significant covariate effect (age or postmortem interval, PMI) was detected; otherwise, one-way analysis of variance (ANOVA) results are presented. P-values for all primary analyses and post hoc tests, corrected for multiple comparisons, are reported: Bonferroni for ANCOVA, Tukey's for one-way ANOVA, and Dunn's for the Kruskal-Wallis nonparametric test. Red text indicates statistically significant findings (* $p < 0.05$). Data shown for female only and male only comparison groups. Abbreviations: *KW*, Kruskal-Wallis statistic; Assays associated with gene symbols are reported in Table S2.

Table S7: Data & Statistics—Medication Comparisons (Associated with Figure 4).

SIGNIFICANT MEDICATION EFFECTS		
**Number of values**	**Mean ± SEM**	**From individual t-tests**
**ADORA2A (MDD: All MDD-NS and MDD-S subjects combined) (Superficial Gray Matter)**		
9 (ON MEDS)<br>4 (OFF MEDS)<br>9 (CTL)	-0.18 ± 0.03 (ON MEDS)<br>0.31 ± 0.23 (OFF MEDS)<br>-0.08 ± 0.07 (CTL)	Welch's t = 2.16, *** P = 0.00 (ON v OFF)**<br>Mann-Whitney U = 24, P = 0.16 (ON v CTL)<br>t (11) = 2.21, *** P = 0.05 (OFF v CTL)**
**NT5E (MDD-S subjects only) (Deep Gray Matter)**		
6 (ON MEDS)<br>4 (OFF MEDS)<br>9 (CTL)	-0.09 ± 0.04 (ON MEDS)<br>0.15 ± 0.05 (OFF MEDS)<br>0.02 ± 0.07 (CTL)	t (8) = 3.54, *** P = 0.01 (ON v OFF)**<br>t (13) = 1.09, P = 0.30 (ON v CTL)<br>t (11) = 1.10, P = 0.29 (OFF v CTL)

Number of values, means, and standard error of the mean (SEM) values for significant purinergic transcripts across cell layers and disease states in the medication analysis. Results from the unpaired two-tailed Student's t-tests are reported. * $p < 0.05$. Abbreviations: *MEDS*, Medication; *CTL*, control; *MDD*, major depressive disorder; *MDD-NS*, MDD–non-suicide; *MDD-S*, MDD–suicide; *ADORA2A*, adenosine A2A receptor; *NT5E*, ecto-5'-nucleotidase.

Supplementary Figures & Captions.

Experiment Design - 384-well qPCR Plate

	1	2	3	4	5	6	7	8	9	10	11	12	13	14	15	16	17	18	19	20	21	22	23	24
A	POOL (SF)	POOL (SF)	POOL (SF)	POOL (W)	POOL (W)	POOL (W)	3	3	3	1	1	1	7	7	7	13	13	13	4	4	4			
B	1:05	1:05	1:05	1:05	1:05	1:05	15	15	15	18	18	18	25	25	25	8	8	8	10	10	10			
C	1:10	1:10	1:10	1:10	1:10	1:10	9	9	9	12	12	12	4	4	4	19	19	19	25	25	25			
D	1:20	1:20	1:20	1:20	1:20	1:20	23	23	23	24	24	24	22	22	22	3	3	3	NoRT (SF)	NoRT (SF)	NoRT (SF)			
E	1:40	1:40	1:40	1:40	1:40	1:40	5	5	5	6	6	6	11	11	11	16	16	16	NoRT (D)	NoRT (D)	NoRT (D)			
F	1 in 80	1 in 80	1 in 80	1 in 80	1 in 80	1 in 80	14	14	14	19	19	19	15	15	15	21	21	21	NoRT (W)	NoRT (W)	NoRT (W)			
G	POOL (D)	POOL (D)	POOL (D)	NTC	NTC	NTC	19	19	19	13	13	13	2	2	2	5	5	5						
H	1:05	1:05	1:05	2	2	2	8	8	8	10	10	10	21	21	21	22	22	22						
I	1:10	1:10	1:10	12	12	12	24	24	24	20	20	20	8	8	8	11	11	11						
J	1:20	1:20	1:20	21	21	21	1	1	1	17	17	17	6	6	6	18	18	18						
K	1:40	1:40	1:40	6	6	6	22	22	22	3	3	3	14	14	14	24	24	24						
L	1 in 80	1 in 80	1 in 80	11	11	11	17	17	17	14	14	14	2	2	2	7	7	7						
M				4	4	4	20	20	20	23	23	23	20	20	20	15	15	15						
N				13	13	13	25	25	25	5	5	5	9	9	9	23	23	23						
O				16	16	16	10	10	10	9	9	9	17	17	17	12	12	12						
P				7	7	7	18	18	18	16	16	16	26	26	26	1	1	1						

Figure S1: Quantitative Polymerase Chain Reaction (qPCR) Assay Experiment Design on 384-Well Plate. Each experiment (plate) was run independently for each primer (*n* = 20). On each plate, three independent standard curves were generated for the superficial gray matter (SF), deep gray matter (D), and white matter (W) cell layers. Light yellow cells indicate the superficial layer tissue, blue cells indicate the deep layer tissue, and green cells indicate the white matter tissue. Negative controls included reactions without cDNA (non-template control, NTC) and reactions without reverse transcriptase (no RT control, NoRT). Each subject was run in triplicate and randomized based on sex and diagnosis. The relative concentrations of the target transcripts (*n* = 16) were calculated independently using a standard curve generated from cDNA dilutions pooled from all subjects for each cell layer. Transcript levels were normalized to the geometric mean of the reference genes (*n* = 4) GAPDH, B2M, ACTB, and PPIA, which were treated as independent qPCR experiments.

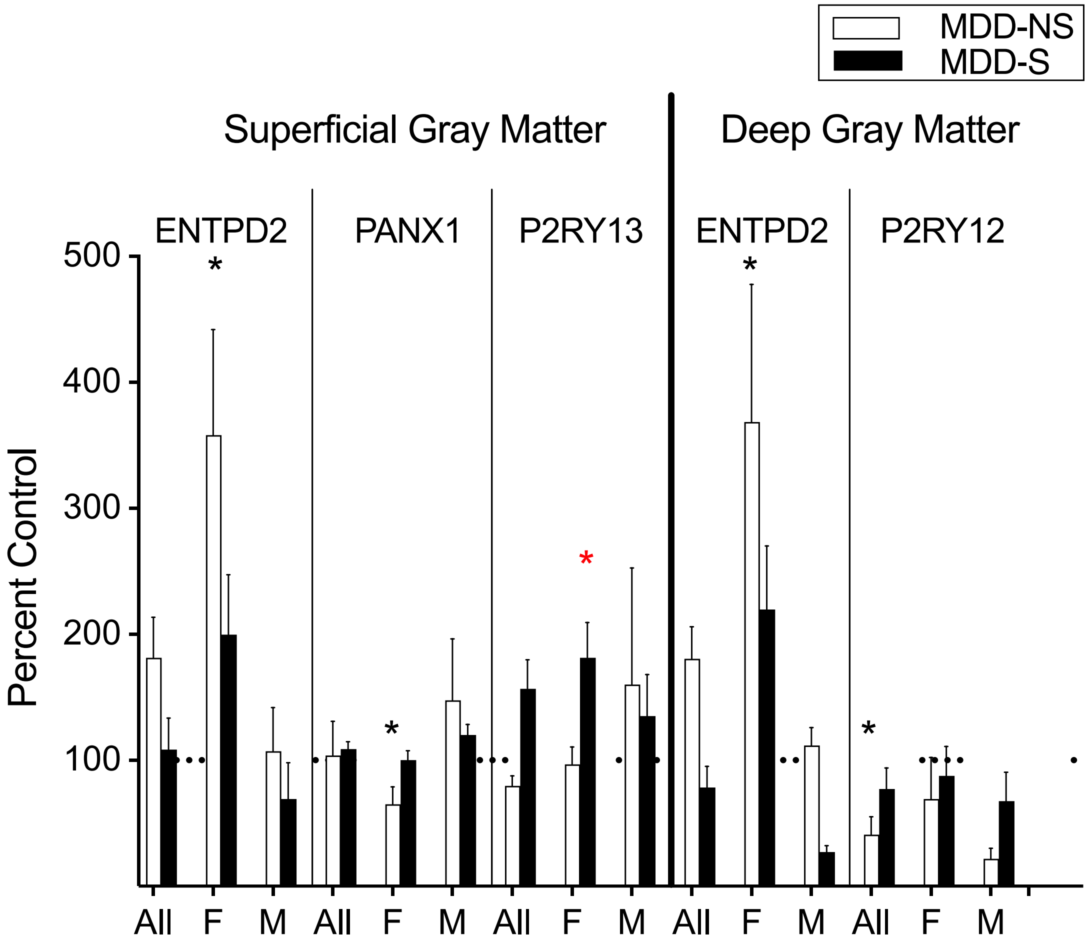

Figure S2: Purinergic Transcript Expression relative to Control groups in the Superficial and Deep Gray Matter. ENTPD2 mRNA expression was significantly greater in MDD-NS vs CTL females in the superficial and deep gray matter. PANX1 mRNA expression was significantly lower in MDD-NS vs CTL females and P2RY13 mRNA expression was significantly greater in the MDD-S vs CTL females in the superficial gray matter. P2RY12 mRNA expression was significantly lower in the MDD-NS vs CTL all subjects group in the deep gray matter. Data reported for MDD-NS (white bars) and MDD-S (black bars) groups as a percentage of the CTL group (dotted line showing mRNA expression for all control groups as 100%). A red star denotes a significant difference in mRNA expression for the MDD-S group relative to the CTL group. A black star denotes a significant difference in mRNA expression for the MDD-SS group relative to the CTL group. Transcripts displayed include those with at least one significant difference between MDD-NS or MDD-S vs CTL groups in at least one comparison group (all subjects, females only, males only). *n* = 3-10 per group. Non-log transformed data was utilized to calculate percentages. Bar graph data presented as mean ± standard error of the mean (SEM). * $p < 0.05$. Data and results of statistical tests are reported in Supplementary Tables 4-5. Abbreviations: *All*, all subjects; *F*, female; *M*, male; *MDD-NS*, major depressive disorder—non-suicide; *MDD-S*, major depressive disorder—suicide; *ENTPD2*, ectonucleoside triphosphate diphospho-hydrolase-2; *PANX1*, pannexin-; *P2RY12*, purinergic receptor P2Y 12; *P2RY13*, purinergic receptor P2Y 13.

Supplementary References.

1. Sullivan CR, Koene RH, Hasselfeld K, O'Donovan SM, Ramsey A, McCullumsmith RE. Neuron-specific deficits of bioenergetic processes in the dorsolateral prefrontal cortex in schizophrenia. Mol Psychiatry. 2019;24(9):1319-28. Epub 20180301. doi: 10.1038/s41380-018-0035-3. PubMed PMID: 29497148; PubMed Central PMCID: PMC6119539.
2. Rosner B. Hypothesis Testing. In: Taylor M, editor. Fundamentals of Biostatistics. Seventh ed. Canada: Brooks/Cole, CENGAGE Learning; 2010.
3. Kane S. Sample Size Calculator. ClinCalc. Available from: https://clincalc.com/Stats/SampleSize.aspx.
4. Dziak JJ, Dierker LC, Abar B. The Interpretation of Statistical Power after the Data have been Gathered. Curr Psychol. 2020;39(3):870-7. Epub 20181002. doi: 10.1007/s12144-018-0018-1. PubMed PMID: 32523323; PubMed Central PMCID: PMC7286546.
5. Siletti K, Hodge R, Mossi Albiach A, Lee KW, Ding SL, Hu L, et al. Transcriptomic diversity of cell types across the adult human brain. Science. 2023;382(6667):eadd7046. Epub 20231013. doi: 10.1126/science.add7046. PubMed PMID: 37824663.
